# Waste Material Utilization in Civil Engineering Applications: Advances, Challenges, and Future Directions—A Scoping Review

**DOI:** 10.3390/ma19143154

**Published:** 2026-07-22

**Authors:** Chathurika Dassanayake, Nuha S. Mashaan, Ridmi Galagedara

**Affiliations:** School of Engineering, Edith Cowan University, Joondalup, WA 6027, Australia

**Keywords:** waste materials, bauxite residue, road pavement, fly ash, tailing, carbon emissions, circular economy, sustainable materials

## Abstract

**Highlights:**

- Reviews key industrial, mining, agricultural, polymeric, and construction wastes in civil engineering.
- Compares material properties, applications, sustainability benefits, and engineering limitations.
- Identifies material-specific suitability across concrete, pavement, and geotechnical sectors.
- Finds variability and lack of standards as key barriers to large-scale implementation.
- Highlights future potential of hybrid systems, digital optimization, and circular infrastructure.

**Abstract:**

This PRISMA-guided scoping review examines the use of waste materials in civil engineering as a sustainable approach to reducing environmental impacts, conserving natural resources, and supporting circular economy principles. The rapid growth of urbanization, industrialization, mining, and agricultural activities generates large amounts of waste materials, including fly ash, ground granulated blast-furnace slag, bauxite residue, mining tailings, waste rock, acid-mine drainage sludge, waste plastics, post-consumer vulcanized rubber, recycled construction materials, and agricultural ashes. The disposal of these materials often creates serious environmental and land-use problems, making their reuse increasingly important. In this context, civil engineering is one of the most promising sectors for large-scale waste valorization because of its high material demand and its ability to use different waste streams into practical applications such as concrete and cementitious systems, pavement and asphalt engineering, geotechnical works, and other infrastructure sectors. This review critically evaluates the global availability, material characteristics, engineering applications, environmental and economic benefits, recent advances, and key challenges related to major industrial, mining, agricultural, polymeric, and construction-derived wastes. Although significant progress has been made in this field, wider implementation is still limited by variations in material properties, technical and environmental challenges, economic constraints, and limited field validation of long-term performance. By bringing together current knowledge from different waste streams and civil engineering sectors, this review highlights important research gaps and future directions to support more sustainable, resilient, and resource-efficient infrastructure development. The effective use of waste materials in civil engineering can play an important role in reducing carbon emissions, improving resource efficiency, and supporting global sustainability.

## 1. Introduction

Civil engineering underpins modern society through developing the world’s infrastructure. The transportation network, buildings, water structures, and other infrastructure are developing within the civil engineering industry and consume significant amounts of natural resources, such as natural aggregates, cement, bitumen, and water. Therefore, the construction sector can be considered as one of the most resource-intensive industries and is majorly associated with energy demand, greenhouse gas emissions, and environmental pollution. The construction industry is a major driver of environmental degradation, contributing approximately 35% of global CO_2_ emissions while also accounting for an estimated 45–65% of landfill waste worldwide [1]. At the same time, due to rapid population growth, industrialization, urbanization, and agricultural development, an enormous quantity of waste is produced worldwide. A substantial proportion of these waste streams is traditionally disposed of by landfilling, stockpiling, or impoundment, resulting in significant health, environmental, and land-use challenges. According to the literature, waste generated in 2017 was 19.8 billion tonnes annually, and it is projected to increase to 28 billion tonnes annually in 2030 and 46 billion tonnes annually in 2050 [2]. Global waste generation is dominated by large-volume waste streams arising from industrial production, mining and mineral extraction, agricultural activities, and construction and demolition sectors [3]. Among these, disposing of the waste generated in industrial and extractive industries, including fly ash, slag from metallurgical industries, bauxite residue from alumina refineries, mine tailings, waste rock, acid mine drainage sludge, agricultural ashes, plastic waste, and construction and demolition debris, poses environmental, economic, and sustainability challenges.

To meet the demands of a growing global population, industrial, agricultural, and other production sectors have expanded substantially, leading to large-scale production of materials and products. For instance, the rate of aluminum production has been rising in line with growing demand, and demand for aluminum production is predicted to grow by 81% by 2050 [4]. The residue produced during the aluminum process, called bauxite residue, is also growing in parallel with the production rate, producing 1.5 to 2.5 tons of residue per 1 ton of aluminum production [5]. Similarly, coal-dependent energy systems continuously produce enormous volumes of fly ash to meet ongoing energy demand. Accordingly, the growing production of waste like Bauxite residue, fly ash, other mining waste, including mine tailing, mine waste rock, and acid mine drainage sludge, requires extensive storage areas and long-term maintenance services because of their high alkalinity, heavy metals, dust generation, and other health and environmental issues [5,6]. Agricultural ashes, including rice husk ash and sugarcane bagasse ash, can pose disposal and environmental challenges caused by their high seasonal generation volumes [7,8]. Waste plastics, including polyethylene, polypropylene, and ethylene vinyl acetate (EVA), are leading environmental issues due to their non-biodegradable nature [9,10,11]. Construction and demolition debris, including recycled concrete aggregate, reclaimed asphalt pavement, and crushed masonry waste, often requires substantial landfill space and thorough management.

Within this context, civil engineering has emerged as one of the most promising sectors for large-scale waste valorization because of its enormous material demand and capacity to incorporate diverse waste streams into functional applications. Depending on the physical, chemical, and mineralogical properties of the waste materials, researchers have made substantial progress in integrating them into civil engineering practice. The diversity of each material is important because it can broaden waste utilization through several replacements for natural materials, such as supplementary cementitious materials, alkali-activated or geopolymer precursors, fillers, aggregate replacements, asphalt binder modifiers, pavement constituents, soil stabilizers, structural fills, or building product components. Utilizing waste materials in the construction industry reduces global landfill demand, natural resource depletion, and carbon emissions, and promotes symbiosis among industries.

Despite the advantages, there are various technical, environmental, economic, and practical challenges and limitations of substituting waste material in construction. Therefore, it is vital to conduct a comprehensive review to critically evaluate waste materials not only by source category, but also by their functional roles across major civil engineering systems. This review examines key waste materials produced in the industrial, mining, plastics and polymers, construction and demolition, and agricultural sectors that are commonly used in civil engineering. The paper assesses their availability, material properties, engineering applications across concrete, pavement, and geotechnical systems, functionality, and sustainability benefits, including a quantitative lifecycle assessment comparison. It also evaluates long-term field performance and durability under real service conditions, discusses existing standards and regulatory limitations, and highlights recent technological advances. Finally, the review identifies current challenges and limitations and recommends directions for future research and standardization. Advancing the effective use of waste materials in civil engineering can support sustainable infrastructure development that contributes to global net-zero targets by 2050 [12] by reducing carbon emissions, improving resource efficiency, and promoting circular economy principles that emphasize waste reduction and long-term material reuse.

## 2. Methodology

**Figure 1:**
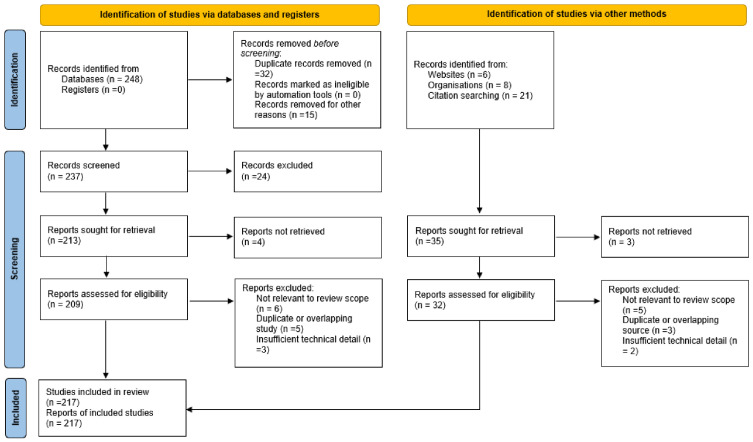
PRISMA flow diagram of the study selection process.

This review was conducted using a PRISMA-guided scoping review framework (see Supplementary Materials), and the purpose of this review is to evaluate the current state of knowledge on the utilization of major waste materials in civil engineering, covering their material properties, engineering performance, sustainability benefits, and practical challenges. Relevant peer-reviewed journal articles, technical standards, government reports, and conference papers from 2014 to 2026 were sourced using the ECU online library and academic databases, such as Scopus, Web of Science, Google Scholar, and ScienceDirect. A combination of the keywords “waste material”, “fly ash”, “GGBFS”, “bauxite residue”, “mine tailing”, “waste rock”, “acid mine drainage sludge”, “waste plastic”, “post-consumer vulcanized rubber”, “biochar”, “rice husk ash”, “sugarcane bagasse ash”, “recycled concrete aggregate”, “reclaimed asphalt pavement”, “pavement engineering”, “soil stabilization”, and “construction” were used to conduct the search, and 217 publications were selected based on their relevance, experimental detail, and applicability in assessing the performance, benefits, and challenges of waste material utilization. The articles were manually screened and reviewed based on title, abstract, full-text relevance, authenticity, and predefined inclusion criteria. Selected studies focused on major waste materials and key thematic areas, including global generation, material properties, concrete and cementitious systems, asphalt and pavement applications, geotechnical applications, sustainability benefits, recent advances, and key challenges. Duplicate, non-relevant, and non-peer-reviewed studies were excluded, and no automation tools were used. This means that, in addition to the main findings, the review also considered basic supporting details, such as the type of waste material, its source, its use in civil engineering, and any important processing methods, while studies with unclear or insufficient information were handled carefully or excluded. Data from included studies were reviewed and organized into consistent thematic categories for comparative synthesis, while unclear, incomplete, or non-comparable information was interpreted conservatively or excluded where necessary. The literature search, screening, eligibility assessment, and final study inclusion are summarized in a PRISMA flow diagram (Figure 1). The review protocol for this scoping review was retrospectively registered on the Open Science Framework (OSF) (https://doi.org/10.17605/OSF.IO/RTNFU).

Table 1 summarizes the characteristics of the studies included in this review, including waste material categories, engineering applications, and representative references.

## 3. Classification of Waste Materials Used in Civil Engineering

**Figure 2:**
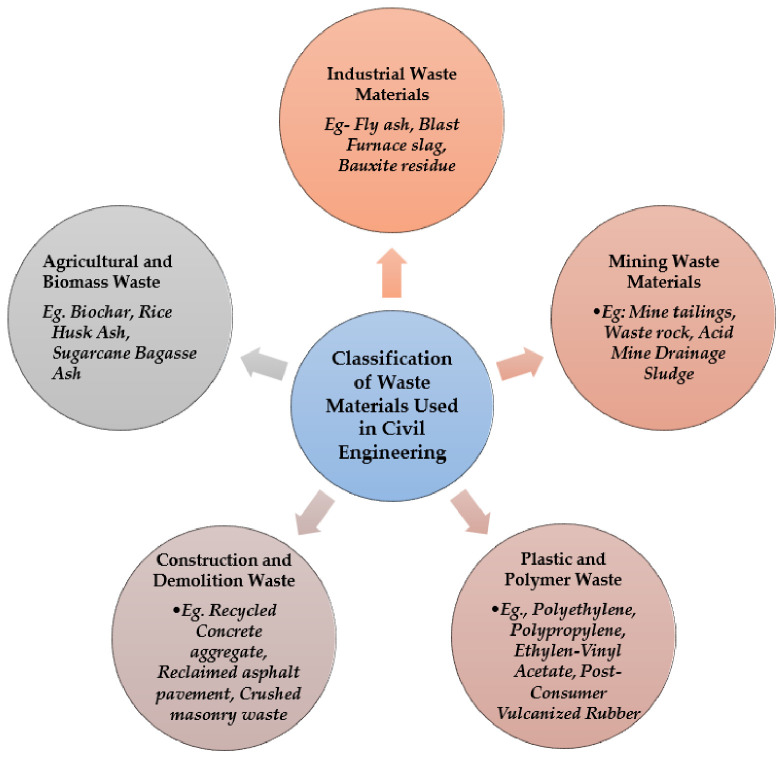
Major categories and examples of waste materials employed in civil engineering applications.

This section classifies major waste materials used in civil engineering industries and describes their global production levels, associated environmental concerns, and key material characterization relevant to construction applications. Figure 2 illustrates the types of waste materials and highlights the most relevant wastes currently used in civil engineering applications.

### 3.1. Industrial Waste Materials

Industrial waste is generated as a byproduct of industrial activities and comprises any material made unusable during manufacturing processes, such as in factories, mills, or mining operations [132]. Industrial wastes consisting of both hazards and non-hazardous components, and the expansion of manufacturing activities and the establishment of industries such as petrochemical, food, oil, and steel, with diverse production, have increased the industrial waste generation [133].

#### 3.1.1. Fly Ash

Fly ash is a byproduct of coal-fired power plants, and global production is estimated at more than 500 million tons per year. Among these, only 25–30% is utilized in various industries [13]. Although coal’s share in the global electricity mix has gradually declined, it remains the world’s largest source of power generation. In 2024, coal-fired power plants produced about 10,700 TWh of electricity, representing roughly 35% of global electricity generation, with most demand coming from the power sector [14]. This continued reliance on coal-fired power plants means that large quantities of fly ash will still be generated worldwide, particularly in rapidly developing regions.

Coal fly ash constitutes approximately 5–20 wt.% of the original coal, occurring mainly as coarse bottom ash and fine fly ash. Of the total ash produced during coal combustion, bottom ash typically accounts for about 5–15 wt.%, while fly ash represents the remaining 85–95 wt.%. Coal ash can be generated through both wet and dry combustion processes. Bottom ash refers to the heavier ash particles that settle at the bottom of the boiler and are mechanically removed. Fly ash consists of finer particles that are carried with flue gases and subsequently collected using electrostatic precipitators or mechanical separation systems [15].

Disposing of the huge volume of fly ash remains a major environmental and health concern. Fly ash disposal is associated with several environmental problems, including air pollution, soil contamination, and surface and groundwater contamination from leaching of toxic elements. Fly ash generated by coal-fired thermal power plants is a significant source of heavy metals, including lead (Pb), nickel (Ni), manganese (Mn), and zinc (Zn). Wind-driven dispersion of fine fly ash particles increases the risk of inhalation, posing potential health hazards [6].

Morphologically, fly ash particles are mainly spherical. The particle sizes are between 10 μm and 50 μm. Fly ash typically has a high specific surface area, ranging from 300 to 500 m^2^/kg. Its bulk density is relatively low to moderate, typically ranging from 0.54 to 0.86 g/cm^3^ [13]. Most of the chemical constituents in fly ash are present in oxidized form, and the material is chemically heterogeneous. The main components are silicon dioxide (SiO_2_), aluminum oxide (Al_2_O_3_), iron oxide (Fe_2_O_3_), and calcium oxide (CaO). These oxides are commonly found within amorphous alumino-silicate and iron-rich spherical particles. Based on the calcium oxide (CaO) content, fly ash is classified into two main types, namely Class C and Class F. Class C fly ash is produced from the combustion of lignite or sub-bituminous coal and contains a high amount of calcium oxide (typically greater than 10%). Class F fly ash is derived from anthracite or bituminous coal and has a low calcium content. Table 2 shows the oxide percentages for fly ash classes F and C [13,16].

#### 3.1.2. Ground Granulated Blast Furnace Slag (GGBFS)

Blast furnace slag (BFS) is a by-product generated during pig iron production in blast furnaces. The slag is produced when the non-ferrous material in the iron, mostly silica and alumina, reacts with calcium and magnesium oxides at temperatures between 1300 and 1600 °C. The produced slag floats on the liquid iron because of its lower density and is periodically tapped from the furnace. When leaving the blast furnace, the slag temperature is around 1450 °C, and it is cooled using various methods. Rapid water quenching produces granulated blast furnace slag with a predominantly glassy structure. This granulated material is then dried and ground into a fine powder to produce Ground Granulated Blast-Furnace Slag (GGBFS). GGBFS is mainly glassy in structure and typically contains particles up to about 5 mm in size [28]. According to studies, worldwide GGBFS production is approximately 530 million tonnes per year, and 65% of it is used in the construction industry due to its latent hydraulic reactivity, which can replace clinker in cement production [29].

The Physical properties of GGBFS are mainly influenced by the cooling method, the grinding process, and the chemical composition of the original slag. The specific gravity of GGBFS is between 2.5 and 2.9, and the bulk density ranges from 1200 to 1670 kg/m^3^, and the surface area varies between 4250 and 4700 cm^2^/g [29]. Further, it can be seen that GGBFS has a mostly amorphous phase and a porous structure, as well as particles with angular and irregular shapes [30].

The chemical composition of blast furnace slag depends on the quality, type, and proportion of raw materials used in the blast furnace, including iron ore, coke, fluxes, and fuels. As a result, slag produced across different regions shows some compositional variation, although it generally remains within a predictable range. Table 3 presents the typical chemical compositions of GGBFS reported in various studies. Granulated blast furnace slag (GGBFS) is primarily composed of calcium oxide (CaO), silicon dioxide (SiO_2_), and aluminum oxide (Al_2_O_3_). It also contains smaller proportions of iron oxide (Fe_2_O_3_), magnesium oxide (MgO), and sulfur trioxide (SO_3_), along with minor trace constituents.

#### 3.1.3. Bauxite Residue (Red Mud)

Bauxite is the main raw material used for aluminum production through the Bayer process. The solid by-product generated during this process is known as bauxite residue, commonly referred to as red mud [42]. With the continued growth of the aluminum industry, the global bauxite residue generation has increased significantly. The Bayer process produces between 1.5 and 2.5 tonnes of bauxite residue per tonne of alumina [43]. The Bayer process has several steps: milling, digestion, clarification, and precipitation. Finally, inside the calciner, the leftover moisture is removed and produces solid alumina (Al_2_O_3_). The solid by-product after digestion and clarification is called bauxite residue [43]. The global bauxite residue generation is approximately 150 million tonnes per annum, and it is estimated that it will increase to more than 200 million tonnes per annum in 2050 [44].

Bauxite residue is considered a waste product containing radioactive material, heavy metals, and high alkalinity (pH 10–12.5) [45,46]. The approaches that directly focus on proper disposal methods and safe reuse are essential to minimizing environmental and health concerns, including soil and groundwater pollution, air pollution from alkaline dust, dam failures, ecosystem damage, and long-term health risks to livelihood.

The physical and chemical composition of bauxite residue varies according to the nature of the bauxite ore and the production process. Table 4 illustrates the typical range of physical properties of bauxite residue. Accordingly, bauxite residue has a higher specific surface area, density, and void ratio.

Iron oxides (Fe_2_O_3_) and aluminum oxides (Al_2_O_3_) are typically the primary components of bauxite residue, although exceptions exist. Additionally, oxides of titanium, silicon, sodium, and calcium are commonly present in bauxite residue. Table 5 shows the typical chemical compositions of bauxite residue reported in different studies.

### 3.2. Mining Waste Materials

**Figure 3:**
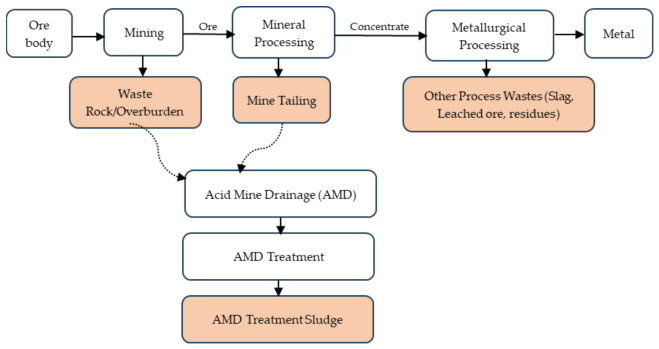
Mining waste generation during mineral extraction and processing.

Mining activities generate large volumes of waste materials throughout the resource extraction process. Figure 3 describes mining waste generation in the process; accordingly, major mining wastes include waste rock and overburden, mine tailings, and other process wastes, such as slag and leached ore residue from the metallurgical process. Further sulfide-rich waste can lead to acid mine drainage (AMD), which in turn produces AMD treatment sludge as a secondary waste stream.

#### 3.2.1. Mine Tailing

Mine tailings are the waste produced after the extraction of valuable minerals and metals from the mine ore. Mine tailings consist of finely ground rock, water, and residual chemicals, which are typically disposed of in tailings storage facilities or tailings dams. The estimated global annual mine tailing production is 7–14 billion tons, generated from the extraction and processing of minerals and metals such as gold, copper, silver, and iron [66].

Mine tailings may contain sulfidic minerals and high concentrations of heavy metals following processing and present a potential environmental hazard. After storage of the tailing, sulfide minerals such as pyrite may oxidize when exposed to rainwater or snowmelt, forming sulfuric acid and leaching the metal from the tailing. Chemicals used in flotation, leaching agents, solvent extractants, and other additives, as well as organics or oils originating from the mining and milling stages, can pose significant environmental hazards within tailings, and contaminant discharge from these sources may adversely impact soils, biota, and downstream water bodies [67]. Therefore, implementing effective recycling methods and utilization is essential.

Generally, the properties of mining tailings, including their physical, chemical, and mineralogical compositions, depend on the ore type, location, and extraction process. Mine tailing particles exhibit angular and irregular morphology due to mechanical crushing and grinding during extraction. Generally, the specific surface area ranges from 0.5 to 7.2 m^2^/g, and porosity values commonly range from 30% to 40%. Further, most of the mine tailing types exhibit 1.8–1.9 t/m^3^ bulk density, and particle density of tailings typically falls in the range of 2.6–2.9 g/cm^3^, depending on the mineral composition of the source [67]. Table 6 presents the typical chemical compositions of various mine tailings reported in various studies.

#### 3.2.2. Waste Rock and Overburden

Waste rock and overburden are major solid wastes generated during mining operations. Waste generated during ore access includes mine waste rock, and preconcentration waste rock is generated when ore is separated prior to mineral processing. Both waste rock and overburden are typically dry and collectively generate over 50 billion tonnes annually [75,76]. With the expansion of mining activities, the volume of waste materials continues to grow, making sustainable management a critical global issue.

The overburden material can be soil, soft or hard rock, and the quantity of material generated can vary according to local geology, mining type, mineral grade, and the technology used in the extraction process [77,78]. Generally, waste rock and overburden are stacked adjacent to the mining area, making waste rock and overburden pile management and maintenance more challenging. Material stacking results in large dumps that are prone to slope failure, compromising production safety and posing risks to human life. Further overburden dumping leads to several significant environmental problems, including loss of biodiversity, water contamination, and land degradation [79].

The chemical and physical properties of the waste rock/overburden exhibit significant variability that depends on the parent rock type. Different mining operations, such as coal, iron ore, bauxite, and copper, produce significantly different types of waste rock. The waste rock generated during mining may contain high levels of sulfide minerals. In Coal mining, the overburden is highly heterogeneous and contains particles ranging from silt to clay-size, as well as large rocks, with variable specific gravity and density. Further, according to the literature, coal mining waste rock has low leachable toxic metal concentrations with permissible limits [80]. Chemically, coal mine overburden is dominated by aluminosilicate minerals, with major oxides including SiO_2_ and Al_2_O_3_, as well as quartz, kaolinite, and other clay phases [81]. The iron ore overburden waste rocks are dense, hard, mechanically stable, and have low water absorption. Further characterized by strong performance in impact resistance, abrasion resistance, and crushing strength. Chemically, iron ore waste consists of Fe_2_O_3_ and SiO_2_ with minor contributions of CaO, MgO, and Al_2_O_3_ [82]. Copper mining waste rocks are highly concentrated with SiO_2_, which is about 78%, with moderate Fe_2_O_3_ and significant sulfur-bearing phases. The waste exhibits moderate density, relatively low porosity and low moisture absorption. Mineralogical component mainly consisting of quartz along with clay minerals, and contains sulfide minerals like chalcopyrite and pyrite [83]. Bauxite overburden is predominantly a fine-grained, clay-rich material with significant variability depending on depth and weathering conditions. This material typically occurs as thick deposits and exhibits a heterogeneous structure, with several mineral phases in the clay. Kaolinite is the main mineral in the bauxite overburden. Goethite and gibbsite are also common, though the amount of gibbsite changes depending on the location and depth. Hematite, anatase, and quartz are found in smaller amounts [84]. Table 7 illustrates the chemical and physical properties of the waste rock/overburden.

#### 3.2.3. Acid Mine Drainage Sludge

Acid Mine Drainage (AMD) Forms when sulfide minerals react with oxygen and water during mining activities and produce iron ferro, sulphate and acid. The process increases the water’s acidity and lowers its pH. Pyrite (FeS_2_) plays a key role in causing AMD, as its oxidation is driven by the presence of oxygen, water, and microorganisms. This highly acidic water has become a significant environmental concern, and treatments are needed. The treatment of AMD has two methods, which are active and passive treatment. Active treatment involves chemical and control systems, whereas passive treatment relies on natural processes [88,89]. Acid mine drainage (AMD) sludge is a secondary waste generated during the treatment of acidic mine water produced by mining activities.

AMD sludge is produced worldwide in regions with active and abandoned mining operations, particularly in coal, gold, and base-metal mining areas. The quantity of sludge generated depends on the acidity of mine water, metal concentrations, and the treatment methods applied. The available data on global sludge quantities are very limited. However, according to the literature, research has been done across 108 operating mine sites; the average AMD sludge production per site is 9500 dry tons per year, with a range of 20 to 135,000 dry tons. And the volume factor is between 2 and 70 times the sludge mass [89].

Although the AMD treatment reduces acidity and metal mobility, the resulting sludge contains concentrations of metals, including Fe, Al, and Mn. As such, AMD sludge poses several environmental concerns due to its composition and large volume, creating challenges for disposal and environmental management. The common environmental management challenges include leaching of contaminants into soil and groundwater, large-scale waste generation, and potential classification as hazardous waste [90].

### 3.3. Plastic and Polymer Waste

Plastics and polymers are used extensively nowadays because they are durable, inexpensive, lightweight, etc. The most abundant polymers used industrially and in everyday life are polyethylene (PE), polypropylene (PP), and ethylene-vinyl acetate (EVA). Plastics are used in construction, packaging, cars, etc. Globally, over 400 million tonnes of plastic are produced every year. Polymers constitute a large volume of waste [93]. Poor management of plastic waste has threatened environmental pollution and endangered sustainability. This section will review the global availability of PE, PP, and EVA, environmental concerns, and their physical and chemical properties.

#### 3.3.1. Polyethylene

Polyethylene (PE) ranks among the largest produced polymers in the world. Polymers are formed from repeating units called monomers. In PE’s case, the monomer is ethylene. These polymers can be classified as low-density polyethylene (LDPE) or high-density polyethylene (HDPE), each with varying properties. PE is generally used in piping, packaging, and construction due to its overall desirable properties, such as toughness, flexibility, and resistance to chemicals and moisture worldwide. The global production capacity of polythene exceeds 100 million tonnes/year. Polythene is non-biodegradable and leads to harmful effects such as landfill waste, microplastics, and marine pollution, endangering ecosystems and human health [94].

Polyethylene has an average density of 0.91–0.96 g/cm^3^ and has a melting temperature between 110 and 130 °C. The impact resistance and ductility of PE are controlled by its semi-crystalline structure that contains both amorphous and crystalline regions. PE has long hydrocarbon chains, which give it its chemical structure. So, polyethylene has high chemical resistance, but it can break down under environmental stresses such as UV exposure [95].

#### 3.3.2. Polypropylene (PP)

Polypropylene (PP) is a thermoplastic polymer. They are produced from propylene polymers. PP has low density, chemical resistance, and moderate strength. These are commonly used in consumer and industrial applications. Global production of polypropylene is more than 80–100 million tonnes annually [135]. PP is mainly used as a disposable product. They accumulate at the end of the lifecycle and are disposed of in landfills because they are non-biodegradable. Also, they form microplastics that adversely affect the environment and humans [94].

Polypropylene has a semi-crystalline structure with a density of 0.90–0.92 g/cm^3^, which makes it the lightest polymer. The melting temperature is 160–170 °C, which is higher when compared with PE. This helps to increase the thermal resistance of the matrix. PP is made of polyhydrocarbon chains (C_3_H_6_) and has high resistance to moisture and chemicals. However, PP degrades under UV radiation. As shown in Table 8, it has the lowest density when compared to PE and EVA.

#### 3.3.3. Ethylene-Vinyl Acetate

Ethylene-vinyl acetate (EVA) is a copolymer made by combining ethylene and vinyl acetate monomers. Vinyl acetate (VA) dictates the properties of EVA, which vary from 10% to 40%. EVA is most commonly used in footwear, packaging, solar panel encapsulation, and adhesives, as it has high flexibility, toughness, and elasticity [136].

Global production of EVA is lower than that of PE and PP. Global production has increased over the past few years [93]. EVA exhibits an amorphous structure compared to PP and PE, as vinyl acetate groups disrupt crystallinity, resulting in high flexibility and low-temperature performance. VA content determines the melting temperature, which is typically between 78 and 110 °C.

Density ranges from 0.93 to 0.95 g/cm^3^, and it has low water absorption properties. The chemical structure contains both polar acetate groups and non-polar hydrocarbon chains, which improve compatibility and adhesion with aggregates in asphalt mixtures. This support is significant in polymer-modified pavement materials [100]. Table 8 and Table 9 show the physical and chemical properties of three waste plastics; accordingly, EVA has a higher density and flexibility compared with PP and PE, showing the copolymer composition.

#### 3.3.4. Post-Consumer Vulcanized Rubber (PCVR)

Post-consumer vulcanized rubber (PCVR) is a rubber product that has gone through its lifecycle after leaving the consumer market and possesses a crosslinked (vulcanized) molecular structure. Most PCVR comes from scrap tires, but lesser amounts come from conveyor belts, seals, hoses, shoes, and other rubber goods. Vulcanization was developed to enhance rubber’s mechanical properties and longevity. The polymer chains in vulcanized rubber are linked by sulfur crosslinks. Vulcanized rubber has desirable properties such as elasticity, chemical inertness, and heat resistance. These crosslinks make recycling/reprocessing more difficult compared to traditional thermoplastics [102].

Millions of tonnes of post-consumer vulcanized rubber are produced worldwide every year. It is estimated that the world produces over 1 billion end-of-life tires (ELT) each year. Recent estimates place ELT production worldwide at over 1.5 billion tires per year. As global transportation needs continue to expand, we can anticipate a sustained surge in waste-tire volumes for years to come [103].

Moreover, this increasing volume of PCVR is also causing significant ecological damage. PCVR products are resistant to biodegradation due to their crosslinked nature and can persist in landfills and natural environments for prolonged periods. When dumped or stockpiled, it can contaminate soil and groundwater reserves, pose severe fire hazards, and cause various types of pollution. Tire fires are notoriously difficult to contain and extinguish once ignited. Toxic chemicals, oils, and particulate matter may be emitted. Microplastics and particulate matter emissions occur due to weathering and the breakdown of rubber products over time [104].

Important properties of PCVR can be attributed to the vulcanization process. Vulcanized rubber is known for being elastic, tough, abrasion-resistant, and weather-resistant. The average density of vulcanized rubber is around 1.10–1.25 g/cm^3^. From a chemical standpoint, discarded tires consist of natural rubber, styrene-butadiene rubber, and polybutadiene rubber, along with carbon black, steel cords, and various additives such as sulfur, zinc oxide, and silica. Carbon black accounts for most of the inorganic material and provides strength. As illustrated in Table 8, PCVR has a complex composition and chemical structure, which contribute to its stability compared to other wastes, such as thermoplastics [105].

Table 8 indicates that PCVR has a higher density (1.10–1.25 g/cm^3^) than PE, PP, and EVA since there are more reinforcing fillers (carbon black, etc.) and additives. Since PCVR is vulcanized rubber, it has a permanently cross-linked elastomeric structure, unlike thermoplastic polymers, and does not have a true melting point. PCVR thermally decomposes at high temperatures. PCVR is flexible, has good chemical resistance, and very low water uptake [103].

As illustrated in Table 9, PCVR’s chemical and structural makeup diverges from that of typical thermoplastic polymers such as PE, PP, and EVA. PCVR comprises a heavily cross-linked elastomeric matrix typically containing natural rubber (NR), styrene-butadiene rubber (SBR), and butadiene rubber (BR), along with sulfur, carbon black, and zinc oxide. Other additives are often used. Vulcanization forms sulfur cross-links that give PCVR superior elasticity, abrasion resistance, and mechanical resilience. The cross-linked nature of PCVR makes it harder to recycle and reprocess compared to PE, PP, and EVA. PCVR cannot be remelted and reshaped by normal thermoplastic recycling processes.

### 3.4. Construction and Demolition Waste

Construction and Demolition Waste (CDW) is the largest contributor to the global solid waste stream. CDW includes a blend of asphalt, concrete, metals, masonry, glass, and wood. These are generated from construction, demolition, and renovation activities. CDW production has risen sharply in recent decades with infrastructure growth, now representing 30–40% of global solid waste, over 3 to 4.5 billion tonnes each year [106,107]. CDW consists mainly of asphalt, concrete, and masonry materials, and most of it can be recycled. Improper disposal of CDW has led to environmental degradation, overuse of landfills, and depletion of natural resources. Also, ecological disturbances such as increased GHG emissions, riverbed erosion, and biodiversity loss can be identified as natural resources are being extracted to replace discarded materials [108].

Characterizing CDW’s physical, chemical, rheological, and durability properties is essential for engineers and researchers to confidently develop sustainable construction practices with long-term environmental benefits.

#### 3.4.1. Recycled Concrete Aggregate (RCA)

Recycled Concrete Aggregate (RCA) is obtained by crushing and processing waste concrete from demolished construction materials. It contains natural aggregates mixed with cement mortar, which considerably affects the material’s behavior compared with raw materials. Residual mortar increases porosity and reduces the density of RCA. Microstructural defects such as weak interfacial transition zones and microcracks can be observed [109,110].

Concrete is the largest contributor to CDW, accounting for 50–70% of total waste generation [106]. As global concrete production exceeds 25 billion tonnes annually, a considerable amount enters the waste stream at the end of service life, continuously supplying RCA [107].

RCA raises environmental concerns mainly due to leaching behavior and chemical composition, as calcium hydroxide and hydration products can affect soil and groundwater chemistry [111]. Furthermore, heavy metals can be released under specific environments. Nonetheless, when compared to natural aggregate extraction, production of RCA has low environmental impacts, where past studies show a 5–8 times lower carbon dioxide emission [112]. The density of RCA varies within 2200–2600 kg/m^3^ as the mortar adheres to it [113]. The high-porosity structure of RCA has resulted in water absorption of 4–10% [112]. Chemical composition is mainly consistent with CaO, SiO_2_, and Al_2_O_3_, with a moderate strength [110]. Table 10 illustrates the physical and chemical properties of RCA.

The rheological properties of RCA are affected by its rough surface texture and water absorption, which increase internal friction and contribute to higher plastic viscosity and yield stress, typically described using the Bingham model. Recent research confirms that high RCA content increases resistance to deformation and reduces flowability due to reduced availability of free water [112]. Finer recycled particles exhibit increased viscosity due to their higher surface area and greater water demand, thereby reducing workability and compaction.

Due to weak interfacial transition zones and porous structure, RCA shows reduced durability. Corrosion and carbonation of the material are enhanced by increased permeability, which allows chlorides, carbon dioxide, and sulfates to enter, accelerating degradation [114]. Residual alkalis contribute to the alkali-silica reaction, and water retention in pores compromises freeze–thaw resistance. Recent studies confirm that treatments such as carbonation can reduce porosity and increase microstructural density, thereby improving the durability of RCA [115].

#### 3.4.2. Reclaimed Asphalt Pavement (RAP)

Reclaimed asphalt pavement (RAP) is retrieved from removing existing asphalt layers or by milling. It contains aggregates coated with aged bitumen, which has a considerable effect on chemical and physical properties due to long-term environmental exposure and oxidation of materials. RAP is widely made CDW material and has recycling rates of more than 80–90% in developed countries, as they have well-established pavement management systems [106].

Polycyclic aromatic hydrocarbons (PAHs) and oxidized hydrocarbons are the primary environmental concerns associated with RAP, as they can be released under specific conditions [111]. Processing and stockpiling also produce emissions and dust, while overall impacts are considered lower than those of extracting the raw materials. RAP has a density of 2300–2500 kg/m^3^ and a smooth, coated structure. Binder content of RAP ranges from 3% to 7% [115]. Even though they exhibit high stiffness, they have low to moderate water absorption. Chemical structure consists of hydrocarbons and minerals. Table 11 outlines the physical and chemical properties of RAP.

Temperature and binder aging affect the viscoelastic behavior of RAP. At low temperatures, oxidation increases stiffness and lowers flowability. Rheological behavior mainly depends on temperature, as the aged binder has higher resistance to deformation and reduced relaxation capacity of RAP [116].

RAP is durable due to the bitumen coating, which limits the moisture access. Oxidative agents lead to brittleness and increased susceptibility to fatigue and cracking. Exposure to environmental conditions may result in binder degradation.

#### 3.4.3. Crushed Masonry Waste

Crushed masonry waste consists of tiles, bricks, and ceramics recovered from demolished structures. It has high compositional variability and exhibits distinct physical characteristics compared to RCA and RAP because it contains clay. Masonry waste accounts for nearly 20–30% of CDW in areas dominated by brick construction [106].

The main environmental issues are dust generation, potential contamination by sulfates (from gypsum), and high water absorption. These can adversely affect air quality and groundwater [110]. Density of Crushed masonry waste is 1800–2400 kg/m^3^ with a high water absorption varying from 8% to 20% [106,116]. Table 12 presents the physical and chemical properties of crushed masonry waste. Due to low strength, they have a brittle structure.

Crushed masonry has poor rheological properties due to its high water absorption and porosity. This results in low flowability, rapid loss of workability, and increased viscosity [116]. The durability of crushed masonry is low due to its high permeability and vulnerability to moisture-related degradation, sulfate attack, and carbonation. Mechanical instability can be observed in weak ceramic structures.

### 3.5. Agricultural and Biomass Waste

Farming and agro-industrial methods create large amounts of agricultural and biomass-based waste. Because of their abundance and environmental impact, rice husk (RHA) and sugarcane bagasse ash (SCBA) have attracted recent attention. Land degradation, air pollution, and greenhouse gas emissions have increased due to improper waste disposal, such as landfilling and open burning.

They differ in physical and chemical properties: they are low-density, high-carbon, silica-containing materials, and often possess porous structures. Properties also vary depending on the processing conditions used and the origin of the raw material. Waste generation, environmental concerns, and some major physical and chemical properties of agricultural and biomass-based wastes will be addressed in this chapter.

#### 3.5.1. Biochar

Biochar is derived from various organic feedstocks such as wood waste, agricultural waste, and manure. Due to its high carbon content, porous structure, and stability, Biochar has gained attention in recent studies. These are produced under low-oxygen conditions via thermochemical conversion of biomass, in general by pyrolysis. The growing availability of biomass waste has increased the biochar production globally [124]. More than 2 billion tonnes of agricultural waste are annually generated. This provides significant feedstock for biochar production. Due to increased attention on carbon sequestration and sustainable waste management practices, biochar production has increased globally [125].

Greenhouse gas emissions and air pollution can increase due to improper management of biomass waste before conversion, such as open burning. If proper control methods are not implemented, biochar production itself can generate emissions [124]. Compared with raw biomass waste, biochar has a reduced environmental impact due to its carbon sequestration potential and stability [124].

Biochar is mainly composed of carbon (50–90%), with smaller quantities of mineral ash, hydrogen, and oxygen. Low density (0.3–0.6 g/cm^3^) and high surface area, more than 300 m^2^/g, can be seen in biochar. The pyrolysis conditions and the type of feedstock govern these chemical and physical characteristics [125]. Table 13 illustrates the physical and chemical properties of biochar.

#### 3.5.2. Rice Husk Ash (RHA)

Rice husk ash (RHA) is a byproduct of rice milling. It is obtained from incinerating rice husks. Of the total weight of harvested rice, nearly 20% is rice husk, making it a substantial source of agricultural waste [128]. In a year, global rice production exceeds 750 million tonnes. 20–25% of rice husk ash is generated during combustion, resulting in millions of tonnes of RHA every year [129]. This can be an opportunity to reuse RHA and provide a positive input to sustainable waste management practices. Land contamination, air pollution, and greenhouse gas emissions can result from the disposal of rice ash in landfills or through open burning. When inhaled, fine ash particles can be a health risk [129].

RHA has a high silica content, generally ranging from 85% to 95%, primarily due to the amorphous form, which depends on combustion conditions. The high surface area is attributed to porous behavior, and the material has a low bulk density of 500–700 kg/m^3^. As shown in Table 14, RHA is primarily composed of silica, distinguishing it from other biomass ashes.

#### 3.5.3. Sugarcane Bagasse Ash (SCBA)

Sugarcane bagasse ash (SCBA) is a byproduct of bagasse incineration. A fibrous residue remains later after extracting sugarcane juice. Brazil, India, and China have large sugarcane production, thus contributing to the widespread production of SCBA [130]. Over 1.9 billion tonnes are annually produced. Nearly 30% sugarcane becomes bagasse, contributing to a large amount of ash in SCBA [131]. Improper management of SCBA can result in dust generation, air contamination, and land pollution. Environmental degradation and health hazards can be exacerbated by uncontrolled burning and open dumping [130].

Table 15 outlines the physical and chemical properties of SCBA. SCBA has a high silica content, nearly 60–80%, as well as oxides such as iron oxide (Fe_2_O_3_) and Alumina (Al_2_O_3_). It has a low density and a high porous structure. Combustion temperature and processing methods define the physical and chemical properties of SCBA [131]. Silica content and other oxides show a heterogeneous composition when compared with RHA. Raw material sources and combustion conditions govern the composition of SCBA.

Table 16 provides a summary of the global annual availability, key material features, and primary environmental concerns associated with all major waste materials classified and discussed in Section 3.1, Section 3.2, Section 3.3, Section 3.4 and Section 3.5.

## 4. Applications of Waste Materials in Civil Engineering

Employing waste materials in Civil Engineering applications is a common practice in promoting sustainability. It helps reduce adverse environmental impacts and protect natural resources. Agricultural waste, industrial waste, mining waste, plastic and polymer testing, construction and demolition waste, and agricultural and biomass waste are widely used to improve material performance in recent studies. These materials reduce landfill disposal and improve their mechanical properties. Material qualities mainly depend on the compatibility with host materials, chemical composition, and treatment methods. So, it is crucial to understand their properties for successful implementation.

### 4.1. Concrete and Cementitious Applications

Concrete and Cementitious Applications are the most common ways to use waste materials in the civil engineering industry, as they require high material demand and environmental impacts. Fly ash, ground granulated blast-furnace slag (GGBFS), and biomass ashes are common waste materials used in concrete applications. These materials have a direct effect on strength development and durability. Waste materials can be used in various applications, such as supplementary cementitious materials, alkali-activated binders, and fillers, promoting the recycling of a wide range of waste materials.

#### 4.1.1. Pozzolanic and Supplementary Cementitious Roles

They vary in physical and chemical characteristics: they are lightweight materials having high carbon and silica contents, and many exhibit porosity. Characteristics will also differ with the processing conditions applied and the source of the original raw material. Waste generation and disposal, environmental issues, and some major physical and chemical properties of agricultural and biomass-based wastes are discussed in this chapter.

Fly ash, RHA, and SCBA are mainly pozzolanic and react slowly with calcium hydroxide and form C-S-H bonds. This will initially reduce strength, but in later stages, strength and durability improvement. GGBFS exhibits latent hydraulic behavior and improves strength and early-age performance in alkaline environments [18,37]. Refining the pore structure in SCM improves permeability and chloride resistance significantly. This helps to handle aggressive exposure conditions. The reactivity and performance of RHA and SCBA solely depend on their processing conditions [56]. Barbhuiya et al. [37] experimented on Ultra High Performance concrete and found that a significant improvement in concrete strength was achieved by using RHA owing to its highly pozzolanic nature. The compressive strength of Ultra-High-Performance Concrete (UHPC) with a 30% RHA replacement level was approximately 182 MPa at 28 days, representing an improvement of about 51% over the control mix. However, an increase in RHA content of more than 30% resulted in a decline in compressive strength, showing that there is an optimum level of replacement [37]. Ahmed et al. [29] found that GGBFS has shown promising strength gains when it replaces some of the cement. Results showed that the highest compressive strength was achieved using a 20% replacement level, raising the 90-day strength from 130 MPa to 140 MPa (an 8% increase) compared to the control mixture. The 56-day strength was also increased from 122 MPa to 135 MPa. Higher replacement levels (>40%) saw a gradual decrease in compressive strength, showing that excessive GGBFS content can be detrimental to mechanical performance despite durability improvements [29].

Golewski et al. [22] found that Fly ash shows higher pozzolanic activity with increasing curing period. The pozzolanic strength activity index (SAI) is low during the initial 3 days, and the strength gain is between 7.4 and 12.5%. A sharp increase in activity is observed between curing periods of 7 and 14 days, with an increase in SAI of over 20%, indicating the onset of significant pozzolanic activity. After 14 days, the increase in SAI decreases, but SAI reaches 92.13% in 28 days. These results confirm the potential of fly ash as a supplementary cementitious material (SCM) for improving the long-term strength of concrete.

A previous study by Javier et al. [140] on sugarcane bagasse ash (SCBA) reported it to be effective as a pozzolanic admixture at a low replacement level. It was reported that mortar specimens blended with 5 wt.% SCBA, used as a cement replacement, exhibited higher compressive strength than those prepared with an ordinary mortar mix. Further addition of silica nanoparticles (Si-NPs) to SCBA mortar significantly improved the performance of the paste/composites due to enhanced particle packing density, accelerated hydration, and the formation of additional C-S-H gel. Addition of SCBA and Si-NPs induced pozzolanic activity and refined microstructure, leading to better strength gain at later ages of curing [140].

Table 17 compares the general SCM behavior/performance of the most significant waste-derived materials applicable as cementitious materials. While all these materials enhance long-term strength and durability through pozzolanic/hydraulic reactions, their early-age performance can differ considerably. Fly ash typically results in slower strength gain development, while GGBFS allows for a more moderate development of strength. Highly pozzolanic RHAs are able to significantly increase strength, and SCBA at low replacement levels has also been shown to perform well, especially when paired with another reactive material. Overall, this shows waste-derived SCMs have the ability to lower cement usage while still achieving desirable mechanical/durability characteristics [37].

#### 4.1.2. Alkali-Activated and Geopolymer Binder Systems

Alkali-Activated and geopolymer binder systems use fly ash, GGBFS, bauxite residue, mine tailings, and rice husks as binders in alkaline solutions. The effectiveness of these materials mainly depends on precursor reactivity. GGBFS shows high reactivity and provides rapid development of strength. Fly ash has a slower reaction rate, which is best suited for long-term performance [19]. Even though Bauxite residue and mine tailings are abundant, they need to be blended with reactive materials because they are low in reactivity. Rice husk ash is a reactive silica resource that improves microstructural densification. They contribute to dense and durable binder systems when designed properly.

Table 18 illustrates the impact of various materials on geopolymer systems, specifically regarding their reactivity. GGBFS has a high calcium content, which contributes to early strength gain along with its faster reaction kinetics. Fly ash is commonly used but requires longer curing times and stronger activation. Fly ash may find application in products that do not require early strength gain [19,37].

Fly ash, bauxite residue, and mine tailings have been shown to have low reactivity when used in binder matrices. Research has consistently shown that these materials need to be mixed with more reactive materials [56]. Rice husk ash has high availability of silica, which helps microstructural densification. This reiterates that geopolymer systems are not necessarily dependent on a single material but perform better when multiple waste streams are combined synergistically. Geopolymer needs to be engineered correctly to function properly.

Slag addition to fly ash-based alkali-activated/geopolymer binder systems has been shown to improve strength development while slightly reducing workability. The addition of 5% slag has been reported to cause only a minor decrease in flowability (8.5%) and to yield a 16% improvement in 28-day compressive strength compared to fly ash-based mixtures. Such improvement has been linked to slag promoting faster reaction kinetics and greater amounts of binder formation, indicating fly ash–slag blends can be successfully used to formulate high-performance alkali-activated binders [23]. The performance of fly ash-based geopolymer systems is strongly influenced by the water-to-binder (W/B) ratio and precursor composition. As expected, higher W/B ratios provide better workability (flowability ranged from 74% to 150%). An increase in the GGBFS ratio results in higher setting times due to its faster rate of C–A–S–H gel formation. Fly ash-based ternary blended system containing GGBFS and metakaolin clay reached 62 MPa at 28 days for compressive strength, which indicates the role of correct precursor combinations in augmenting the geopolymerisation process and matrix densification [39].

Activated with alkaline solutions, Bauxite residue has demonstrated promise as a geotechnical stabilizer. NaOH-activated soil–BR mixtures have exhibited notable UCS gains after 7 days of curing (2.23–3.05 MPa), outperforming many BR-stabilized soils tested in other studies (UCS values ranging from 0.31 to 2.15 MPa after 28 days of curing) [46]. They attributed this excellent performance to alkali activation, which resulted in the formation of cementitious reaction products and high strength gains of the treated soils [46]. These findings suggest that BR has the potential to be used as a sustainable additive for pavement and ground improvement applications [141].

Mine tailings have also been shown to possess great potential as alkali-activated/geopolymer precursors, often when used in combination with slag. The results indicated geopolymer blends containing 70 wt.% tailings achieved strengths of around 31 MPa at 28 days, while optimal geopolymer brick mixtures reached strengths of 12.8 MPa, 32.4 MPa and 49 MPa at 7 days, 28 days, and 90 days curing time, respectively. Copper leaching was also found to be low (between 0.5 and 2.4 mg/kg), below set regulatory limits and tailings were shown to immobilize toxic leachates, showing promise for mine tailings-based geopolymers as a sustainable construction material for bricks and other civil engineering applications [58]. In alkali-activated binder systems, RHA has also shown promise as an alternative precursor material. Due to its inherently high silica content, rice husk ash has been used as the source of silica in alkali-activated geopolymer binder systems. Compared to fly ash-based geopolymer systems, geopolymers containing RHA showed a higher liquid demand as a result of smaller particle sizes associated with RHA. Additionally, compressive strength increased by increasing activator concentration and with an optimized RHA/activator ratio, with values over 34 MPa observed under optimal conditions and over 29 MPa across a range of optimized mixes. These strengths were attributed to better dissolution of silica and alumina during geopolymerization [142].

#### 4.1.3. Filler, Fine Aggregate, and Partial Replacement Roles

Beyond reactive SCMs, numerous waste materials are added to concrete primarily for their physical (as opposed to chemical) properties. Bauxite residue, mine tailings, acid mine drainage (AMD) sludge, crushed masonry waste, and recycled concrete aggregate (RCA) are all common examples of materials that often make most of their contribution as filler, fine-aggregate replacement, or low-reactivity mineral additions. These additions are primarily controlled by particle size distribution, shape, and texture rather than pozzolanic or latent hydraulic activity.

The primary effect for most of these materials is packing. Finer particles fill gaps between larger grains, allowing for a denser particle packing, which could decrease overall porosity and improve durability if a well-designed mix is created. The reality is, however, that many of these wastes (especially bauxite residue and mine tailings) have high specific surface areas and angular particles, which increase the water demand of the mix. In the absence of adjustments to the mix design (e.g., adding a superplasticizer), workability is reduced, which can lead to increased porosity during hydration [57,59].

Recycled concrete aggregate and crushed masonry waste can add further issues as well. Due to the mortar attached to these particles, both are more porous than natural aggregates and absorb more water. Both have been shown to decrease compressive strength and increase its variability (negatively), particularly at higher replacement rates. [110,119]. AMD sludge has been examined rarely as a brick/block filler, which similarly allows for waste stabilization while being utilized as a construction material [91].

Bauxite residue (red mud) has been considered as a potential partial replacement material for cement and fine aggregate in concrete. While optimum performance has been found to be at about 10% replacement in most studies, yielding compressive strengths similar to or greater than those of conventional concrete mixes, replacement levels exceeding 10–15% typically result in inferior strength. Moderately adding bauxite residue to concrete can increase durability while allowing for responsible waste utilization [143]. Mine tailings have been used as replacements for fine aggregates in concrete mixes. Optimum replacement ratios are usually found between 10% and 35%, but replacement levels up to 60% have produced similar compressive strengths to control mixes. The improved performance is thought to result from the filler effect of the addition of fine tailings particles, thereby improving matrix packing. Increases in replacement level have been found to decrease workability and increase water demand [60]. Due to the presence of RCA, the overall mechanical properties of concrete decrease. Researchers observed that when 20% of the cement was replaced by fly ash, there was a 6.75% improvement in compressive strength after 28 days. However, increasing RCA replacement ratios from 15% to 60% reduced compressive strength and flexural strength values by approximately 12–46% and 7–20%, respectively, at 28 days. These decreases in mechanical performance can be attributed to the increased porosity of recycled aggregate and a weak interfacial transition zone [144]. Crushed masonry waste has also been studied as a partial replacement for fine aggregate. Replacement ratios of 10–20% have been shown to provide acceptable mechanical performance, with compressive strengths at 28 days between 27 and 29 MPa for crushed brick aggregates. These strengths are adequate for the requirements of M25 grade concrete. Above 25% replacement ratios, there is a considerable drop-off in compressive strength, amounting to around 44–50% reduction at 50–75% replacement. Optimum replacement ratios are thus believed to lie at 15–20% [145].

The downside of using these wastes is often an expected decrease in mechanical properties. However, the advantages of avoiding landfill disposal fees, conserving natural aggregates, and decreasing overall material costs can outweigh these decreases, especially for structures where high strength is not required. Table 19 summarizes the key properties and functional effects of waste materials used as filler or aggregate replacements in concrete.

Although using filler and aggregate replacement materials can reduce mechanical properties, many waste materials can achieve adequate strength when replacing aggregate at an optimal percentage. Many offer the ability to divert waste from landfills while reducing the use of virgin aggregate, creating more sustainable and affordable concrete. Table 19 summarizes the performance of these materials along with some limitations. Improved packing potential was noted for bauxite residue and mine tailings, but there was usually another drawback, such as high surface area. This goes to show again how important gradation and mix optimization are. RCA and crushed masonry waste are usually more directly linked to increased porosity and decreased strength [57]. AMD sludge is one of the least researched materials on this list, but it is included to show the direction research is taking in using problematic waste streams as BC [91,95].

However, the table also shows that they typically suffer from reduced strength. This drawback is usually not severe enough to outweigh the benefits of their use, given their eco-friendliness. Ideal for uses where strength is not heavily needed.

### 4.2. Pavement and Asphalt Engineering

Road construction’s substantial material requirements make using waste an immediate opportunity to create circular economy flows and achieve high impact. Waste polymers, industrial by-products, and recycled aggregates can be used in asphalt binders and mixtures for pavement engineering applications to enhance desirable properties such as rutting resistance, stiffness, and durability while mitigating adverse environmental impacts. When used in pavement applications, these materials can act as modifiers to asphalt binders, mineral fillers, or aggregate substitutes, and can be used in any layer of a pavement structure. When integrating these materials, it is crucial to thoughtfully assess their compatibility, moisture resistance, and long-term performance.

#### 4.2.1. Binder Modification with Waste Polymers and Carbonaceous Additives

**Figure 4:**
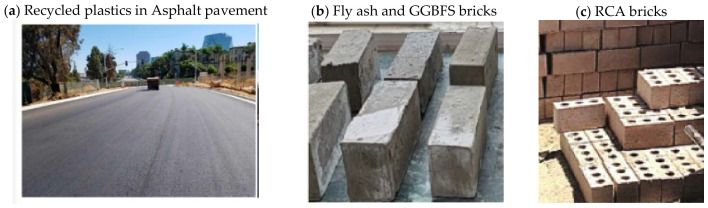
Representative civil engineering products incorporating waste materials: (**a**) asphalt pavement modified with recycled plastics [154], (**b**) fly ash and GGBFS masonry blocks [155], and (**c**) recycled concrete aggregate (RCA) bricks [156].

There has been increasing interest in modifying asphalt binders with waste-derived polymers and carbonaceous materials to produce roads with improved pavement performance while simultaneously addressing plastic waste and biomass-derived challenges. Polyethylene (PE), polypropylene (PP), ethylene-vinyl acetate (EVA), biochar, and bauxite residue, among others, are materials that are being evaluated for use in bitumen modification for enhanced mechanical and rheological properties (Figure 4a).

Wet and dry processes are the two ways polymers can be combined with asphalt binders. The wet process involves adding polymers to hot bitumen before mixing in aggregates. The dry process consists of adding polymers at the time of mixing with aggregates. The dry process of mixing polymers into asphalt is less complex than wet processing, but leaves polymers less evenly dispersed [10].

Ensuring compatibility and maintaining phase stability present a core difficulty when working with polymer-modified binders. Low compatibility can arise between bitumen and polyolefins such as PE or PP, leading to phase separation when stored over time. Because of its polarity, EVA exhibits greater compatibility when mixed into the binder matrix [101].

Performance-wise, all modifiers significantly improve resistance to rutting by enabling stiffer compounds at higher temperatures. This increase in stiffness is often accompanied by a loss of flexibility at low temperatures, making roads more prone to cracking. Biochar has been identified as a potential additive, with porous structures and high carbon content, showing promising aging resistance and binder stability [126]. Table 20 illustrates the summary of the behavior of waste material during binder modification.

Post-consumer vulcanized rubber (PCVR) is often utilized as crumb rubber or ground tire rubber (GTR) modifier in asphalt binders and asphalt mixtures. PCVR possesses a highly cross-linked elastomeric polymer network structure that can effectively improve the rheological performance of asphalt binders, such as elasticity, viscosity, and resistance to permanent deformation. Experimental research found that rubberized binders typically demonstrate superior rutting resistance and fatigue life compared to that of unmodified asphalt binders at high service temperatures. Rubber-modified asphalt binders exhibit a high dependence on particle-rubber interactions affected by particle size, rubber content, blending conditions, and digestion time. As rubber particles are mixed into the asphalt binder, lighter components of the asphalt are absorbed by the rubber particles, which swell during the mixing process, leading to a modification in binder stiffness and viscoelastic response. On the other hand, high rubber content usually leads to higher mixing and compaction temperatures and poorer storage stability due to phase separation. Regardless, using PCVR to modify asphalt binders and mixtures is an excellent solution for reducing the large number of end-of-life tires that end up in landfills each year while increasing the long-term performance and durability of asphalt pavements.

Recycled EVA and HDPE increased the rutting resistance of asphalt binders, with softening point increases of about 25% and 19%, respectively. Penetration was decreased by 43–46% by both polymers. EVA had better elastic recovery and fatigue resistance, while HDPE resulted in a stiffer binder with less resilience [146].

Modification with crumb rubber (CR) can drastically improve asphalt performance; asphalt with CR can resist rutting up to 7 times better, be around 5 times less sensitive to aging, and show up to 12.6 times better fatigue life. With high CR levels, the softening point increased by about 22%, indicating improved high-temperature deformation resistance [147]. Biochar has demonstrated potential as a sustainable asphalt modifier, as evidenced by reported increases in binder stiffness, rutting resistance, and aging resistance. Results included a 32–35% decrease in penetration, an increase in critical rutting temperature from 65.2° C to 70.1° C, and a decrease in aging index from 43.5% to 36.5%, which indicated potential for longer-lasting pavement performance [148]. Bauxite residue (red mud) has shown potential as an asphalt filler, with 3–15% incorporation improving softening point, viscosity, and rutting resistance. However, higher red mud contents reduced ductility and low-temperature cracking resistance while enhancing moisture resistance and aggregate–binder adhesion.

As shown in Table 20, polymer modifiers such as PE and PP are quite adept at enhancing rutting resistance. Still, incompatibility with bitumen poses a challenge for their extensive implementation [10,101]. EVA has been reported to be more stable, but it may not be affordable. Post-consumer vulcanized rubber (PCVR), often used as crumb rubber or ground tire rubber, has also been studied as an asphalt modifier. Like other elastomers, PCVR can impart high rutting resistance to asphalt binders, as well as improved resistance to low-temperature cracking. PCVR differs from PE and PP modifiers in that it improves the binder’s elasticity and fatigue performance, rather than merely increasing its stiffness. Increased binder viscosity and storage stability issues, due to phase separation between the asphalt binder and rubber, may also occur with PCVR.

Biochar, as an emerging modifier, not only exhibited improved mechanical performance but also exhibited superior aging resistance, suggesting it could improve the long-term performance of pavements [126]. Despite the limited literature, bauxite residue could serve as a supplemental modifier to enhance the stiffness of the asphalt binder. Generally, no single material can meet all performance requirements, and further investigation is needed to balance stiffness, flexibility, and aging stability.

#### 4.2.2. Mineral Filler and Aggregate Replacement in Asphalt Mixtures

Fly ash, GGBFS, bauxite residue, crushed masonry waste, recycled concrete aggregate (RCA), and mine tailings are examples of waste materials used as mineral fillers/additives or as aggregate replacements. The incorporation of these wastes affects mechanical performance due to their interactions with the binder and aggregate matrix.

Marshall stability indicates the load-carrying capacity of the mixture. Angular and rough-textured wastes can increase stability because particle interlocking improves; examples include crushed masonry and slag [20].

Moisture susceptibility is another important concern, as mentioned earlier. Because it is highly porous, RCA has a greater capacity to absorb both binder and water. This can lead to increased stripping potential and lower durability. Mine tailings may also experience inconsistent performance because of their mineralogy and gradation [61].

Filler–binder interaction affects both stiffness and rutting resistance. Fly ash and bauxite residue are ultrafine particles that can increase binder stiffness. However, using too much could also make mixtures brittle, so a proper mix design is required. The angularity and absorption properties of aggregates also affect mixture performance.

Fly ash has shown promise as a substitute for mineral filler in asphalt mixtures. The optimum bitumen content decreased from 5.21% to 4.8–4.9% at fly ash replacement levels of 4–6%, and Marshall stability increased from 13.56 kN to 15.44–19.48 kN. The maximum stability achieved was 19.48 kN at 6% fly ash. This shows better strength and resistance to deformation than conventional fillers [24]. Ground granulated blast furnace slag (GGBFS) could be a potential mineral filler for asphalt mixtures. The optimum GGBFS content was 3% by weight of the mixture, increasing Marshall stability from 16.16 kN to 19.16 kN (18.6% increase) while maintaining a flow near 2.8 mm. These results suggest higher rutting resistance and better mechanical properties when compared to traditional limestone-filled asphalt mixtures [40]. Research has found that bauxite residue (red mud) can be used as a replacement mineral filler in asphalt mixtures. Permanent deformation decreased from 6.10% with conventional filler to 3.50–5.31% when red mud was used as filler at replacement levels of 3–7%, demonstrating enhanced rutting resistance. However, asphalt mixtures with red mud required a slightly higher optimum asphalt content (5.66% compared to 5.34%) and showed a decrease in moisture resistance, as shown by retained Marshall stability values decreasing from 88.57% to 82.28% [149].

Recycled concrete aggregates have been successfully used to replace natural aggregates in asphalt mixtures. Recommendations for RCA content are usually up to 30% because, above this replacement level, the optimum asphalt content increases by about 0.5–1.0% due to the porous nature of RCA. Performance has shown both improvement and reduction. Marshall stability increased by up to 45%. However, air voids have increased at high RCA contents, leading to decreased moisture resistance and indirect tensile strength. RCA can provide usable pavement performance while preserving natural aggregates [150]. Mine tailings could be sustainable sources of asphalt fillers and improved stiffness, softening point, viscosity, and rutting resistance. The softening point of asphalt mastic was increased by 14.6% by the addition of red mud. Permanent deformation decreased from 6.10% to 3.50–5.31% upon adding tailings at replacement levels of 3–7%. Except for softening point tests, tailings decreased ductility and moisture resistance values, meaning they would need further treatment or anti-stripping additives to ensure long-term pavement durability [62]. Table 21 illustrates the performance of waste material as a mineral filler and aggregate replacement.

From Table 21, it can be concluded that most of these waste materials can substitute traditional fillers/aggregates without compromising engineering properties and thus can be used in asphalt mixtures. Fly ash, GGBFS, red mud, RCA, and mine tailings showed good enhancement in stiffness/rutting resistance, but minor concerns, such as increased binder demand and reduced moisture resistance at higher replacement levels, were observed with some materials. Overall, these studies confirmed the feasibility of using these waste materials in asphalt mixtures, paving the way towards sustainable pavements with less dependence on virgin construction materials.

#### 4.2.3. Recycled Asphalt and Hybrid Pavement Systems

Recovered asphalt pavement (RAP) is the most common recycled material in asphalt mixtures due to its high sustainability and economic advantages. The aged binder present in RAP is stiffer and more brittle than virgin bitumen. Despite improved rutting resistance, aged binder has negative impacts on fatigue and cracking resistance [117].

Various rejuvenation techniques have been implemented to restore binder flexibility, including the addition of softening agents, virgin binder, or modifiers such as PE, PP, and EVA. Biochar could also enhance binder performance and mitigate aging.

RAP can be used in conjunction with other recycled materials, like RCA or crushed masonry, in layers to create a hybrid pavement system. When developing hybrid pavements, engineers are guided by circular economy principles, intending to maximize the reuse of materials without a loss of structural strength. Typically, as RAP content increases, so does the stiffness of a mixture. Excessive stiffness in a pavement system can cause early-age cracking [116]. Table 22 summarizes the roles, major benefits, key challenges, and performance impacts of recycled asphalt and hybrid waste material systems in pavement engineering applications.

RAP has been shown to perform well in asphalt mixtures when the RAP content exceeds 20%, and appropriate modification techniques are used. It was observed that only 45–60% of the RAP binder contributed to the blend with the virgin binder; the average blending efficiency was 56.5%. In asphalt mixtures with 50% RAP content, only 20–28% of the binder was active in blending. The addition of rejuvenators and warm-mix asphalt technologies, with optimized binder content, has been shown to enhance rutting, fatigue, and moisture resistance, allowing higher percentages of RAP to be used in sustainable pavement construction [120]. Ground tire rubber (GTR), also referred to as post-consumer vulcanized rubber (PCVR), can greatly enhance the performance of asphalt binders, including increases in elasticity, rutting resistance, and creep recovery [121]. The addition of 20% GTR decreased penetration by 17.2%, increased softening point by 18.7%, shear strength by 45.9%, and increased creep recovery at high stress levels to 80.8%. Negatively, ductility decreased by 30.9%, while mixing and compaction temperatures increased by about 29% and 27%, respectively. Mixing and compaction temperatures increasing drastically can lead to poor workability. Optimum GTR content is usually recommended to be between 10% to 20% to achieve improved performance without sacrificing constructability [122].

In general, waste plastics enhance the high-temperature performance of asphalt through increased stiffness, viscosity, and rutting resistance, while negatively affecting low-temperature flexibility. Plastic-optimum contents range between 3 and 8 wt.% depending on the polymer used. Of all polymers tested, LDPE incorporated at 10 wt.% provided the best rutting resistance, whereas 5 wt.% PP caused up to a 20% reduction in ductility. Additional levels of 2–4 wt.% EVA resulted in better rutting and low-temperature performance, while HDPE enhanced stiffness and moisture susceptibility while decreasing resilience. PET-based additives improved rutting resistance by at least 15% and fatigue resistance by up to 60% when used in RAP mixtures [121].

Table 22 presents the performance review of asphalt pavements of RAP-based hybrid systems. The addition of RAP shows a positive effect on stiffness and rutting resistance. However, modifiers such as GTR, waste plastics, and biochar improved the binder’s durability and aging resistance. Hybrid systems with RCA or crushed masonry can enhance sustainability, although proper optimization is needed to achieve both mechanical performance and moisture resistance.

### 4.3. Geotechnical Engineering Applications

Incorporating waste materials into geotechnical projects such as ground improvement, earthworks, and pavement support offers a prime opportunity for their effective use. Because these applications often require large volumes of materials, industrial wastes, and construction wastes such as fly ash, slag, mine tailings, and recycled aggregates are potential alternatives to natural materials that can yield environmental and economic advantages if suitable for soil improvement. These materials can help reduce soil compressibility and swelling or improve strength if treated properly or combined with native soils. It is necessary, however, to assess their mechanical performance, moisture characteristics, and environmental risks, such as leaching and acid-generation potential, before concluding on their suitability for long-term dependable use.

#### 4.3.1. Soil Stabilization and Improvement

Fly ash, GGBFS, bauxite residue, acid mine drainage (AMD) sludge, and mine tailings are some examples of waste materials employed in soil stabilization processes to enhance the engineering behavior of soft subgrades. These amendments generally improve strength properties via pozzolanic reactions, particle bonding, and modification of the original soil fabric.

Fly ash and GGBFS rank among the most efficient stabilizers owing to their cementitious behavior. Treating fly ash with lime or cement has demonstrably led to substantial gains in UCS and CBR, while also effectively lowering the plasticity and swelling tendency of expansive soils. GGBFS has shown excellent strength gain at early ages due to its latent hydraulic properties [38]. Bauxite residue (BR), also known as red mud, is another industrial byproduct that has been utilized for soil stabilization purposes. Studies have shown that due to its alkaline nature, BR has sufficient pozzolanic activity and can be used for stabilization. However, environmental concerns such as pH requirements and the potential for toxic heavy-metal leaching may arise, and proper investigation must be carried out prior to use [26,119]. AMD sludge has also been used to enhance soil strength characteristics. However, similar to BR, there is a potential risk of contamination.

Tailings are abundant globally and have been found to improve soil density and strength upon blending with a binder. As with most waste materials discussed, their effectiveness depends on their gradation and mineralogy, and they often require chemical activation or mixing with cementitious materials to show promise [61]. Table 22 summarizes the effectiveness of waste materials used in soil stabilization, highlighting their influence on strength improvement, swell reduction, environmental concerns, and activation requirements.

Fly ash has been extensively used to stabilize expansive soils because of its pozzolanic nature and effectiveness in reducing plasticity, swelling, and compressibility. The optimum contents of fly ash to stabilize expansive soil varied based on various soil types and classes of fly ash used. The expansive soil characteristics improved up to 40% replacement level. At this replacement level, compressive strength was improved by 40–48%, swelling by 42–48%, compressibility by 36–40%, and CBR by 52–55%. Improvement of expansive soil with Class C fly ash showed better improvement compared to the Class F fly ash due to the higher calcium content of Class C fly ash. Blending of fly ash with GGBFS, lime, or cement improved the strength and CBR of soil by further pozzolanic reactions [25]. Bidirectionally activated GGBFS modified with an optimal 2.5% phosphogypsum (PG) content increased the UCS of the stabilized soil by around 40% due to the improved formation of cementitious products. A bidirectional activator system reduced the cost of stabilization by 43%, carbon emissions by 37%, and carbon emissions per unit of compressive strength by up to 70% compared to traditional cement-based stabilization [41]. Thermally activated BR stabilized dispersive soils. UCS and BTS values increased by up to 426% and 167%, respectively, with optimum BR content of 2%. Calcium carbonate formation and hydration reactions increased strength gain through improved particle bonding and stabilization over a 28-day curing period [151]. Mine tailings (MT) were found to be effective in stabilizing expansive soils. The addition of 20% MT and 4% lime increased UCS from 40.1 to 260.5 kN/m^2^ and reduced the liquid limit from 58.4% to 43.2% (37.1% with lime addition). Maximum dry density increased from 15.8 to 16.3 kN/m^3^ while optimum moisture content decreased from 25.9% to 24.0%. Shrinkage limit increased from 8.4% to 18.4% [63].

Table 23 shows that waste materials can significantly improve soil engineering properties through pozzolanic and cementitious reactions. Fly ash, GGBFS, bauxite residue, and mine tailings all greatly increase strength and decrease plasticity and swelling potential. AMD sludge, however, is better used to stabilize contaminated soils. These wastes benefit the environment by offering sustainable replacement stabilizers and minimizing waste disposal issues.

#### 4.3.2. Embankment and Structural Fill and Cover System Applications

Waste rock, mine tailings, AMD sludge, crushed masonry waste, and recycled concrete aggregate (RCA) have been extensively used in embankments and structural fills. These materials have been selected mostly based on availability, compaction behavior, and shear strength. Waste rock and mine tailings can meet compaction and shear strength requirements for embankment construction if proper gradation and moisture content are achieved. However, if the waste rock contains sulfide minerals, there is potential for acid generation, leading to long-term durability issues [85].

Crushed masonry waste and RCA are frequently used because they typically have good strength and angularity, which lead to better interlocking characteristics. However, they have higher porosity than natural soils, which can affect their hydraulic behavior [110,119]. AMD sludge is not commonly used in embankments, but it has been studied in cover systems for its potential to immobilize contaminants. Long-term performance and environmental safety of AMD sludge should be carefully considered. Table 24 summarizes the engineering performance of waste materials used in embankment and fill applications.

Magnesite mine tailings (MMT) have shown promising performance as a sustainable structural fill material. By increasing the relative density from 25% to 75% and the embedment depth to 1B, the ultimate bearing capacity (UBC) and the modulus of subgrade reaction increased from 150.5 to 762.6 kPa and from 26,140 to 90,960 kN/m^3^, respectively. Leaching tests showed acceptable environmental risk if properly contained, proving MMT to be both a sustainable, environmentally friendly, high-strength alternative fill material [64]. Recycled concrete aggregate (RCA) performed admirably as a subgrade stabilizing agent as well. Substituting 20% of natural soil with RCA resulted in a 6.35% increase in maximum dry density, a 175.7% increase in UCS, and a 175.5% increase in shear strength. Fatigue life also increased by 10–55%, while CBR values decreased by 4.1%. Overall, these findings suggest that limited RCA replacement can improve the mechanical performance and longevity of pavement subgrades while lessening the need for natural resources [152].

Table 24 showed that certain materials made from waste materials can be used as alternatives to traditional embankment/fill materials. The MMT had good bearing capacity and stiffness, and RCA improved subgrade strength and fatigue life with negligible loss in CBR. Crushed masonry and AMD sludge can potentially be used as fill materials, but their engineering performance is highly dependent on material quality and proper management of exposure to water/leaching.

#### 4.3.3. Base and Sub-Base Layer Applications

Waste materials commonly used in pavement base and sub-base layers include waste rock, RCA, reclaimed asphalt pavement (RAP), GGBFS, and mining tailings. These materials help contribute to the bearing capacity/resilient modulus needed to support traffic loads. RCA and waste rock tend to exhibit adequate strength and load-distribution characteristics, allowing them to be used directly in base layers [86]. Tests showed that the Waste Crushed Rock (WCR) specimens reinforced with 0.5–2% of crumb rubber exhibit sufficient shear strength to meet the criteria for use as pavement base and subbase materials. The addition of rubber improved the cohesion (25.3–41.6 kPa) and increased the internal friction angle and shear stiffness due to better particle interlocking. Despite the observed trend of decreasing cohesion with increasing rubber content, all specimens exceeded the minimum shear strength needed for pavement base and subbase materials [87].

Based on past studies, cement-treated recycled concrete aggregate (RCA) has great potential for use in pavement base and subbase applications. Mixtures containing ≥3% cement met the minimum 7-day UCS of 2 MPa, as specified by Austroads and TxDOT. Also, 5% and 7% cement satisfied UCS specifications for low- and high-volume roads of 3 MPa and 4.5 MPa, respectively. Mixtures containing ≥2% cement had weight losses less than 14% after 12 wet–dry cycles, meeting pavement specification requirements [153]. RAP material has shown good potential for use as a granular sub-base. Using RAP mixed with natural aggregates increased the soaked CBR value from 32% to over 100%, with an optimum RAP blend level of 55% when used as a sub-base course. An optimum RAP content of 50–55% was suggested by previous studies to achieve acceptable strength and durability properties with minimized use of natural aggregates [123]. Based on past research, fly ash treated with lime and GGBFS emerged as highly promising options for pavement base materials. An optimum mix of fly ash, lime (3%), and GGBFS (9%) met the strength criteria for the base layer. The lime- and GGBFS-treated fly ash sample shows better results due to the development of cementitious hydration gels, which proves that fly ash can replace natural aggregates and increases pavement sustainability performance [27]. The suitability of zinc mine tailings as road construction materials and structural fill was investigated. They presented a dry density of 18.62 kN/m^3^, a CBR of 11%, a friction angle of 34°, and a modulus of 18.17 MPa. They displayed adequate strength characteristics for road construction materials, including load-bearing capacity and stiffness. These properties were enhanced through cement stabilization. This material could be used for pavement sub-base applications, with potential benefits of decreasing aggregate thickness by about 170 mm [65].

Experimental results presented in Table 25 indicate that some waste materials can be effective substitutes for traditional materials used in pavement base and sub-base layers. Cement-treated RCA and RAP, fly ash–GGBFS mixture, and zinc mine tailings met the necessary strength and durability requirements for pavement use. Rubberized waste-crushed rock increased shear resistance by better interlocking.

### 4.4. Other Applications of Waste Materials

Construction materials and products for masonry applications represent one of the largest opportunities for the use of waste in civil engineering. Bricks, blocks, tiles, and lightweight panels can all be produced incorporating significant proportions of fly ash, bauxite residue, mine tailings, and recycled aggregates to replace natural materials and with acceptable properties. These materials affect strength, water absorption, density, and thermal properties. As is typical with most of these products, a trade-off exists between mechanical performance and thermal insulation. Nevertheless, bricks, blocks, and panels made from waste are widely used in non-structural and semi-structural masonry.

#### Building Materials and Masonry Products

Fly ash, bauxite residue, mine tailings, recycled concrete aggregate (RCA), and crushed masonry waste have all been used to manufacture bricks, blocks, tiles, and lightweight construction panels. Since these masonry products are non-load-bearing, their properties often vary over a wider range than those of structural concrete, allowing greater flexibility for waste incorporation.

Fly ash bricks and blocks have shown compressive strength comparable to or greater than that of traditional clay bricks due to enhanced particle packing and pozzolanic reaction products [13] (Figure 4b). Mine tailings and bauxite residue may also enhance compressive strength through stabilization processes or blending with cementitious binders [26,61], but exhibit greater variability due to mineralogical differences among the waste materials. Water absorption of masonry products is typically lower than that of structural concrete, as it is undesirable for durability reasons; however, bricks and blocks made from RCA and crushed masonry inherently have higher water absorption due to their porous, broken particles and attached mortar [110,119] (Figure 4c).

Density-wise, the addition of waste materials typically yields lighter products (particularly in lightweight panels). This decrease in density is advantageous for decreasing dead loads of buildings and increasing ease of handling during construction. In addition, most provide improved thermal properties, insulation value increases with porosity and lower density. The relationship between strength and thermal insulation is often inversely proportional: materials with lower density typically provide enhanced insulation but reduced compressive strength. Thus, care should be taken when choosing materials based on whether they will be used in structural applications.

## 5. Environmental, Economic, and Sustainability Benefits

The environmental, economic, and sustainability benefits of utilizing waste in the engineering industry are significant. The reuse of the above discussed material reduces reliance on virgin raw materials and minimizes the environmental burdens associated with them. Those significant benefits drove research and industry interest in the reuse of waste-derived materials across various construction applications.

The construction industry is one of the largest contributors to environmental degradation, generally generating nearly 35% of global CO_2_ emissions and 45–65% of landfill waste [1]. The use of waste materials in the construction industry helps mitigate environmental impacts by reducing cement consumption and minimizing waste disposal. Cement production is a major source of greenhouse gas emissions, and it is increasing at a 2.5% annual rate. Generally, cement production generates 1350 million tonnes of greenhouse gases per year. This includes emissions from fuel combustion, limestone decomposition, and electricity consumption. In addition to those, this requires 1500 kg of raw material and 80 units of electricity per tonne [1]. Hence, the partial replacement of cement with waste materials such as fly ash, ground granulated blast-furnace slag, rice husk ash, and sugarcane bagasse ash can significantly reduce energy consumption and emissions. For example, according to Orozco, et al. [157], replacing 50% of cement with fly ash reduces the global warming potential (GWP) from 357 kg CO_2_ eq/m^3^ to 164 kg CO_2_ eq/m^3^, a reduction of about 54%. Further replacing cement with 65% of ground granulated blast furnace slag reduced emissions by nearly 141 kg CO_2_ eq/m^3^, which is 61% reduction. Similarly, the use of recycled aggregates reduces emissions associated with quarrying and transportation. Agricultural residues, including rice husk ash (RHA) and sugarcane bagasse ash (SCBA), also provide significant environmental benefits when used as partial replacements for cement. Studies have reported reductions in global warming potential (GWP) of up to 25% for RHA and 32% for SCBA [158,159,160]. Similarly, using biochar at low replacement levels of 3–5% can reduce greenhouse gas emissions by 12–20% [161,162]. In addition to cement replacement, recycled concrete aggregate (RCA) helps lower emissions by reducing the need for quarrying natural aggregates and transporting raw materials, with GWP reductions of up to 64% compared with natural aggregates [163,164]. In asphalt construction, reclaimed asphalt pavement (RAP), crumb rubber, and waste plastics can be used as bitumen modifiers or aggregate replacements, reducing the demand for virgin aggregates and petroleum-based binders and thereby lowering emissions. Likewise, using bauxite residue as a partial cement replacement has been reported to reduce GWP by approximately 26% [165].

Table 26 summarizes the environmental and economic benefits of key waste materials based on available life cycle assessment and cost analysis data reported in the literature.

Another major benefit is reduced landfill disposal. Huge quantities of waste materials, such as fly ash, mine tailings, and construction and demolition waste, can be removed from their disposal locations and reused in civil engineering applications. This helps to reduce land degradation and long-term environmental risks. For instance, fly ash dumps in ash ponds have been reported to require large land areas, and their disposal not only causes land degradation but also air, water, and soil pollution. The reuse of mining waste materials, particularly tailings, offers significant environmental benefits by reducing the need for large-scale storage facilities and associated land degradation. According to the literature [169], tailings storage facilities (TSFs) are extensive engineered systems that occupy vast land areas and pose long-term environmental and stability risks. By diverting tailings from these storage systems into construction applications, the amount can be substantially reduced. Furthermore, the use of mining waste reduces the risk of environmental contamination, including soil and groundwater pollution due to the leaching of hazardous elements from tailings deposits. Reducing reliance on tailings storage facilities also lowers the likelihood of catastrophic failures, which have been identified as a major global environmental concern. Similarly, bauxite residue requires a significant land area for storage facilities, commonly managed through lagooning and dry stacking methods. Lagooning involves storing the residue in a large containment pond as a slurry, which requires continuous monitoring to prevent leakage and environmental contamination. Though dry stacking is considered a safer method than lagooning, both methods lead to land-use challenges and potential soil and groundwater impacts, and require long-term sustainable management requirements. Therefore, utilizing bauxite residue in the construction industry significantly reduces the large storage challenges and mitigates associated risks [5].

The use of waste materials in civil engineering significantly reduces demand for natural resources by replacing conventional materials such as cement, aggregates, and bitumen. Industrial by-products such as fly ash and ground granulated blast-furnace slag (GGBFS) can replace a substantial portion of Portland cement, typically 15–30% of the mass of OPC in a concrete mixture by fly ash. Similarly, GGBFS can replace cement, typically around 30–50%, also with higher levels of 30–85% reported in the literature. Similarly, agricultural ashes such as rice husk ash and sugarcane bagasse ash are commonly used as partial cement replacements, generally between 5% and 20% [7,8,29,170,171]. Replacing cement with waste materials reduces the consumption of natural raw materials such as limestone and clay, as well as the energy required for clinker production.

Mining-related waste, including mine tailings, waste rock, overburden, and bauxite residue, can substitute for natural aggregates or fillers in concrete and pavement applications at varying levels, in some cases up to full replacement of natural aggregates. For example, according to previous studies, iron tailing is able to replace 10–20% pavement base, 74% coarse aggregate, 12% fine aggregate,40% as sand, and 5% as cement in concrete [172,173,174,175]. Similarly, recycled concrete aggregate (RCA) and reclaimed asphalt pavement (RAP) reduce the need for virgin aggregates, with RAP typically used up to 30% in pavement construction and partially replacing the demand for virgin bitumen in the mixture [176,177]. The replacement of natural aggregates with waste materials reduces quarrying and sand mining activities, thereby conserving natural resources and mitigating environmental impacts. In addition, plastic wastes such as polyethylene, polypropylene, and ethylene vinyl acetate, as well as biochar, can partially replace bitumen or conventional modifiers, reducing demand for petroleum-based materials. Acid mine drainage sludge and other mining wastes can replace clay in brickmaking and be used in treatment systems, helping keep waste out of landfills. Overall, adding these waste materials to construction saves resources, mitigates pollution, and supports a circular economy [9,10,11,90,127]. Table 27 summarizes the various waste materials and the natural materials they replace in construction applications, highlighting their potential to reduce the consumption of virgin resources.

Utilizing waste materials in the civil engineering industry offers economic advantages by reducing material and waste-disposal costs and enhancing life-cycle performance. The economic benefits of utilizing waste materials depend strongly on local availability, processing requirements, transport distance, and performance requirements.

The use of fly ash in construction materials yields financial benefits by reducing cement consumption and overall construction costs. Additionally, fly ash-based masonry processes can reduce embodied energy consumption by up to 90%, demonstrating significant cost efficiency [170]. According to Paruthi, et al. [188], selected optimized blends of fly ash and GGBFS can achieve a material cost noticeably lower than that of traditional burnt clay bricks. Further, according to the cost analysis conducted in that study to quantify the economic benefit of fly ash-GGBFS–lime binders, fly ash-rich blends reduce binder costs compared to cement-only systems. Francioso, et al. [159] conducted a cost analysis to assess the economic impact of using sugarcane bagasse ash as a partial replacement for cement, and the study showed that at a 30% replacement, the cost of mortar can be reduced by up to 18.5%. The cost reduction is primarily attributed to the low material cost of SCBA, which is significantly lower than that of Portland cement. Further reuse of SCBA reduces landfill disposal costs, providing additional economic advantage. Although the process incurs additional transportation and processing costs, the net cost of the overall process remains lower, demonstrating the economic viability of SCBA as an alternative supplementary cementitious material in construction. Similarly, GGBFS, rice husk ash, and mine tailings can partially replace cement, which is one of the most expensive components of concrete.

The use of natural aggregate replacement materials, such as reclaimed asphalt pavement (RAP), recycled concrete aggregate (RCA), and waste rock, reduces the need for quarrying and mining of sand and associated processing costs. Waste-derived materials, such as waste plastic, EVA, and biochar, contribute to cost savings for petroleum-based binders by acting as bitumen modifiers or fillers. Biochar is considered cost-effective because it can reduce construction costs while being produced from low-cost waste materials. Studies have shown that the production cost of biochar ranges from about $222 to $584 USD per ton, and this cost can be reduced further when the energy generated during production is utilized. In construction applications, incorporating biochar has been reported to reduce mortar costs by approximately 13–25 US $/m^3^. Additionally, even small additions of biochar (around 2%) can lead to about a 1% reduction in cement cost, contributing to overall economic savings [127].

In addition to direct material cost reductions, the utilization of industrial and agricultural wastes such as bauxite residue, mine tailings, and acid mine drainage sludge helps avoid landfill disposal and handling costs, providing further economic advantages. The continued rise in mining waste, including bauxite residue generation, has resulted in rising costs for storage, land, and environmental management. According to Wang, et al. [189], the construction cost of the bauxite residue storage facility at the Shandong alumina plant is approximately $15 million, and its service life is only up to five years. In addition, the annual maintenance and management costs exceed approximately USD 2 million, further increasing the long-term financial burden. The cost of landfill disposal for bauxite residue is also considerable, typically estimated at USD 10–20 per ton, with an average handling cost of approximately USD 15 per ton [189]. Therefore, the reuse of bauxite residue in construction applications offers a practical approach to reducing disposal costs and long-term economic burdens. Although those waste materials may require processing for reuse or recycling, transportation, the net financial gain is generally positive due to the combined effect of reduced raw material consumption and avoided waste management costs. Therefore, incorporating these waste materials supports cost-effective construction practices while promoting the circular economy principles. The approach to waste material utilization in the construction industry leads the industry towards greater resource efficiency, lower environmental impact, and more sustainable practices, supporting long-term resource management.

## 6. Long-Term Field Performance and Durability of Waste-Based Materials

Although laboratory investigations have demonstrated the performance of waste material utilization across different sectors of civil engineering, there remains limited evidence of long-term field performance under real-world service conditions. The majority of existing studies rely on short-term performance data and predictions, which can vary widely depending on field exposure, such as environmental exposures, traffic conditions, seasonal variations, and other unavoidable factors.

### 6.1. Field Performance of Waste-Based Cementitious Systems

Long-term performance of fly ash-modified cementitious systems has been investigated in several studies through notable field exposure programs. Pasupathy, et al. [190] investigated the 10-year long-term performance of fly ash-based geopolymer concrete, which was exposed to a severe salt lake environment, and compared the results with OPC concrete that had the same exposure conditions. The finding reveals that fly ash-based GPC exhibited greater susceptibility to chloride penetration, while its diffusion coefficient was nearly 1.5 times higher than that of OPC concrete. The corrosion rate of the OPC concrete is less than that of the GPC concrete. Another important observation was the greater loss of alkalinity in GPC. The pH of GPC showed a range of 7.0–8.0 at the surface to the reinforcement level, whereas OPC concrete maintained a pH value between 12.0 and 12.5. Similarly, a field exposure condition study by Wang, et al. [191], demonstrated that high-performance concrete containing 15–35% of fly ash dosage and 10% of fume exhibited superior corrosion resistance at a water-binder ratio range between 0.24 and 0.38 after 8 years under actual field conditions in the Qinghai Salt Lake region of China. The outcome of these studies highlighted the importance of long-term field validity, as an accelerated laboratory test may not capture the full extent of real environmental conditions, and the replacement level of waste material can be misleading without specific site conditions.

Ground granulated blast-furnace slag (GGBFS) also has a well-established record of improving concrete durability in aggressive environments. The study by Tahwia, et al. [192] demonstrated that concrete containing blast-furnace cement, which is rich in GGBFS, showed better resistance to sulfate attack compared to OPC after 180 days of exposure to a severe sodium sulfate solution. The GGBFS concrete exhibits minimal strength deterioration and a dense microstructure. These findings support the long-term durability potential of GGBFS concrete in sulfate-rich service environments. A comprehensive review of geopolymer concrete [193] reported that waste-derived aluminosilicate materials, including industrial and agricultural by-products, provide superior durability compared with conventional Portland cement concrete. Geopolymer concrete exhibited enhanced resistance to sulfate attack, acid exposure, chloride penetration, and elevated temperatures due to its stable geopolymer binder and dense microstructure.

For agricultural waste-containing cementitious applications, evidence of field performance is substantial. The laboratory investigations confirmed that materials such as RHA and CBA substantially improve the concrete resistance to chloride ingress, carbonation, water absorption, and acidic environment. Concrete with 10% RHA and SCBA showed lower permeability, thereby increasing resistance to corrosion-related deterioration compared to normal concrete [194]. However, long-term field exposure studies and monitoring of structures incorporating RHA and SCBA remain limited, highlighting the need for real-service validation to confirm their durability performance over extended periods.

### 6.2. Long-Term Performance of Waste Materials in Pavement Engineering

In pavement engineering, recycled concrete aggregate (RCA) is among the waste materials that have been extensively evaluated for performance under field conditions. Cheng, et al. [195] investigated the re-cementation behavior of RCA in a heavily trafficked highway in Hong Kong, which has used RCA for pavement sub-base for 13 years. When old concrete is crushed to produce RCA, the process breaks it into pieces. But some of the original cement in that concrete was never fully consumed when it first hardened, and it is still chemically active. When this recycled aggregate is placed in a road sub-base, rainwater and groundwater seep in. That leftover active cement then reacts with water, just like fresh cement does, and starts to harden, binding the loose aggregate particles together. The results showed that most of the selected sampling locations have experienced re-cementation, and the stiffness of the RCA layer increased steadily over the last period. The stiffness gain tends to enhance the cracking resistance and permanent deformation. However, severe re-cementation increases the risk of reflective cracking. These findings demonstrate that long-term performance uncertainties are not captured in laboratory investigations.

A real-world performance evaluation of plastic-modified asphalt was conducted by Ma, et al. [196], and a trial road pavement was constructed in 2022 in Newtonville, Canada. Using mixed waste plastic and polyethylene terephthalate (PET) fibers at a dosage of 0.3–0.6% in both the surface and binder courses. Core samples collected after construction were tested to assess rutting and cracking resistance. The results showed that PET fibers improved resistance to rutting at high temperatures and reduced crack propagation at low temperatures, whereas mixed waste plastics produced more variable results depending on temperature. However, the authors noted several limitations, including the use of an overdesigned asphalt binder, the high natural sand content of the mixture, which may increase freeze–thaw damage, and the fact that the cores were tested shortly after construction, before significant binder aging had occurred. Therefore, the study recommended continued field monitoring and testing of aged pavement cores to better understand the long-term performance of recycled plastic-modified asphalt.

Further, studies have shown the promising performance of crumb rubber-modified asphalt under real field conditions. Sierra-Carrillo de Albornoz, et al. [197] evaluated asphalt mixtures containing crumb rubber from end-of-life tires on heavily trafficked highway sections over a monitoring period of up to 63 months. The study found that the density of the asphalt layers remained stable throughout the service period, while the stiffness and fatigue resistance of crumb rubber-modified mixtures were comparable to those of conventional SBS-modified asphalt. The results indicated that crumb rubber asphalt exhibited similar aging behavior and mechanical performance under severe traffic and climatic conditions. These findings provide valuable field evidence that crumb rubber can be successfully used in asphalt pavements, while also offering an environmentally sustainable solution for recycling waste tires.

### 6.3. Environmental Durability and Leaching Considerations

An important aspect of the long-term field performance of waste-based construction materials is the potential release of contaminants over time. Although short-term leaching tests are commonly used to assess environmental safety, the behavior of these materials under actual field conditions may be different because of factors such as rainfall, carbonation, and acidic environments. The long-term leaching behavior of waste materials used in construction is an important environmental issue that is still not fully understood. In practice, fly ash has a strong alkaline buffering capacity, and its behavior can change over time due to environmental processes such as carbonation and acid rain. As a result, these short-term tests may underestimate the potential for long-term heavy metal release. Li, et al. [198] investigated groundwater contamination caused by fly ash in road base materials. The study used accelerated carbonation testing together with reactive transport modeling and Monte Carlo simulations. The study revealed that although fly ash initially exhibited low heavy-metal leaching, as carbonation progressed, heavy-metal release increased. The range increased from 5.6 to 1073 times higher than the initial level. Also, studies have shown that the contamination risk level varies with hydrogeochemical conditions, highlighting that short-term tests are insufficient to assess environmental safety.

Similarly, Diotti, et al. [199] studied recycled construction and demolition materials and found that sulfates, copper, and some heavy metals could exceed environmental limits depending on the source material. The study also showed that carbonation and pH changes can influence the release of contaminants from recycled aggregates over time. Materials containing gypsum or certain concrete components were found to be more susceptible to contaminant release.

The use of mining waste in road construction can create long-term environmental problems, especially related to soil and groundwater pollution. The study by Xu, et al. [200] investigated the environmental risks associated with the use of iron tailings as road subgrade material through leaching experiments. The study’s findings indicate that elements such as arsenic, manganese, barium, nickel, and lead may be present. Specifically, in areas with higher humidity, groundwater concentrations of magnesium and nickel may increase, exceeding permissible levels during rainfall. Overall, the results of the study indicate that the environmental behavior of the mine tailings strongly depends on the climate and requires site-specific long-term environmental risk assessment prior to the application of road infrastructure.

Overall, these studies suggest that the environmental performance of waste-based construction materials may change during their service life. Environmental conditions such as acid exposure, rainfall, and carbonation can affect both contaminant leaching and material endurance. Therefore, long-term environmental monitoring, together with mechanical performance assessment, is important to ensure the safe use of waste-based materials in construction applications.

## 7. Challenges and Limitations

Although there are significant sustainability and economic benefits associated with using waste materials in civil engineering, limitations and challenges still hinder their large-scale use.

One of the major limitations is the high variability in the properties of each waste material. The physical, chemical, and mineralogical properties of the same type of waste material are influenced by the production conditions of the source material from which it is derived. For example, the properties of mining waste, including mine tailings, waste rock, overburden, and bauxite residue, are strongly influenced by ore type, extraction process, location, weathering conditions, and storage environment. Table 2, Table 4 and Table 5 clearly defined the variability in the chemical composition of GGBFS, bauxite residue, and mine tailings across different studies. The variability makes it difficult to develop a universal mix design, identify the optimum replacement percentages, and ensure consistent performance specifications. Similarly, variability in fly ash quality due to differences in coal sources affects its performance and reliability in construction applications. Further, construction waste, including RCA, crushed masonry, and RAP, the performance of the replacement depends on the source concrete or masonry items, such as grading, contamination, and attached mortar content.

Another major issue with using waste material is health and safety concerns, which should be carefully evaluated and managed. Mining waste, including mine tailings, bauxite residue, and Acid mine drainage sludge containing potentially hazardous substances, including heavy metals and alkaline compounds. Bauxite residue is highly alkaline (pH > 13) [5], which requires pretreatment, such as heat treatment and neutralization, before engaging with construction materials. Further, the presence of potentially harmful elements (e.g., As, Cr, V, Pb) highlighted the leaching concern, while NORM (naturally occurring radioactive material), particularly U and Th, requires radiological assessment of bauxite residue before application [5]. Acid mine drainage sludge also contains concentrated precipitated metals and residual acidity. When using bricks or as cementitious materials, proper material characterization and leaching analysis are required prior to reusing [89]. The long-term leaching behavior of these materials under real service conditions is examined further in Section 6.3. These types of factors, engaging with each waste material, necessitate careful evaluation and limit their large-scale application in construction.

Technical performance limitations must be carefully considered when using waste materials in construction. Although materials such as fly ash and GGBFS are widely used to develop low-carbon binder systems, particularly in geopolymer and alkali-activated materials, their performance depends heavily on factors such as the type of activator, curing conditions, replacement level, and chemical composition. For example, strength and setting time can be adversely affected by improper curing or by the selection of an unsuitable activator. Excessive replacement levels may negatively affect performance, such as reducing workability, increasing water demand, and raising durability concerns. For agricultural wastes such as RHA and SCBA, the burning temperature, fineness, and unburnt carbon content strongly affect the final product performance. For instance, the study Al-Alwan, et al. [201] highlights that improper combustion of rice husk can lead to the formation of crystalline silica and a high carbon content, which negatively affect concrete performance. Furthermore, the particle size of RHA highly affects its effectiveness. The larger particles can reduce strength, while finer particles improve performance by filling pores and increasing density. The results also indicate that increasing the percentage of RHA content beyond an optimal level yields only marginal strength gains, underscoring the importance of optimizing replacement levels. These findings highlight that although waste materials can enhance the performance of construction materials, their effectiveness is highly dependent on technical performance, including controlled processing, proper mix design, and optimal material proportions.

One major challenge is the lack of consistent standards for mix design, material processing, and performance evaluation when reusing waste materials. Section 6 covers how certain waste streams perform over time in the field, but without unified, performance-based guidelines, it is difficult for the industry to use these materials with confidence. As a result, industry adoption remains low, even though research findings are promising. To help make waste materials safer and more widely used in civil engineering, it is important to create clear, material-specific standards. While some of the established waste products in construction benefit from dedicated standards, the situation is much less so for newer and alternative waste-derived materials. The usage of fly ash in concrete is covered by ASTM C618, which classifies fly ash as class F and C based on its chemical composition and sets requirements for properties such as fineness, strength activity, water demand, soundness, and autoclave expansion and contraction [202]. In Europe, EN 450-1 [203] sets out similar requirements, including limits on loss-on-ignition and chemical composition. Ground-granulated blast-furnace slag is regulated by ASTM C989 [204] and EN 15167-1 [205], which classifies slag according to its reactivity and performance characteristics. The availability of these standards has helped the construction industry adopt these materials with confidence, as they are supported by long-term field performance and established mix design practices. Further, ASTM D6114 [206] is a specification for asphalt rubber binder prepared with ground recycled rubber and other additives. The standard specifies the dosages, blending conditions, and the binder’s performance properties.

ASTM C1709 [207], the standard guide for the evaluation of alternative supplementary cementitious materials provides a framework for assessing materials not covered by existing specifications, but it functions as an evaluation guide rather than a prescriptive specification and requires project-specific demonstration of performance. Accordingly, the utilization of other waste types, including bauxite residue, mining tailings, waste rock, acid mine drainage sludge, waste plastics, recycled construction materials, and agricultural ashes, should undergo a case-by-case evaluation for each waste type and application. Recycled aggregates are covered by EN 12620 [208], but their use is still limited. There is no international standard available for agricultural wastes that specifically addresses agricultural waste ashes as cementitious materials, despite their demonstrated pozzolanic potential in laboratory studies. Hence, developing a comprehensive standard for each material based on its conditions and prioritizing long-term field validation data collection are essential for the safe and widespread use of waste materials in the civil engineering industry.

Economic and logistical barriers are also significant challenges in the use of waste materials in construction. In most cases, waste materials are not readily available at the construction site, and transporting them over long distances can significantly increase the project’s overall cost. For example, most mining sites are located in remote or regional areas, far from the construction market, significantly reducing their economic viability. In addition, most waste materials require pre-processing, including drying, grinding, heating, and chemical treatment, to ensure health and safety and performance. The processes may increase the complexity and cost of reusing. Therefore, the real sustainability benefit depends not only on replacing natural aggregates, cement, bitumen, or fillers, but also on processing energy, transport emissions, local availability, and regulatory acceptance.

Overall, the main limitations are material variability, health and safety risk, technical performance limitations, lack of standardized specifications, limited field performance data, and uncertain economic feasibility. These issues show that waste material utilization should be based on source-specific characterization, appropriate treatment, performance-based mix design, and long-term environmental assessment.

## 8. Recent Advances in Waste Material Utilization

Recent advances in waste material utilization in civil engineering have moved beyond simply replacing conventional materials. Current research is receiving growing attention on engineered waste-based materials, hybrid modification, advanced testing, digital optimization, and performance-based design.

A major breakthrough is the development of hybrid material systems. Instead of using a single waste stream, recent research combines two or more waste streams to offset their individual weaknesses. For example, studies have found that fly ash can be blended with GGBFS to improve the compressive strength and workability [209], while char can be combined with plastic wastes, including polyethylene and polypropylene, to enhance asphalt binder stiffness, aging resistance, and thermal stability [210]. In pavement materials, integrated systems using RAP, recycled aggregates, biochar, plastics, and polymer modifiers are gaining attention because they enable both mechanical improvements and greater waste incorporation. Recent studies have shown that low-reactivity waste materials such as ultrafine mine tailings can be effectively incorporated into engineered ternary geopolymer systems. For example, the incorporation of GGBFS into tailings–fly ash systems considerably enhances geopolymerization through the formation of C-(A)-S-H gels, causing improved strength and densified microstructure [211]. While hybrid material systems are commonly explored in research, recent studies further advance this approach by developing practical mix-design frameworks for multi-stream, waste-based concrete. For instance, the study by Tejas and Pasla [212] has been successfully designed to achieve structural-grade strengths (40–80 MPa)of alkali-activated recycled aggregate concrete incorporating agricultural waste ash (sugarcane bagasse ash and rice husk ash) and GGBFS. Such approaches provide engineers with reliable design models while improving interfacial bonding and overall advanced performance of recycled concrete systems.

Recent advances also include improved processing and pre-treatment techniques. Waste materials are increasingly processed through grinding, sieving, thermal treatment, carbonation, chemical activation, washing, neutralization, and surface modification before use. For example, Zhang, et al. [213] successfully used biomass-derived biochar as a low-carbon reducing agent to treat bauxite residue, improving metal recovery and modifying its physicochemical properties. The porous structure and high surface area of biochar facilitate gas diffusion and reaction rates, enabling efficient reduction under regulated thermal conditions. Biochar properties are also strongly influenced by pyrolysis temperature, residence time, and feedstock type [214]. While rice husk ash and sugarcane bagasse ash require controlled burning to increase their amorphous silica content and loss on ignition (LOI), recent studies have shown that even high LOI ashes can be successfully utilized through appropriate bending and advanced processing strategies [215]. Recent studies are focusing on tailoring these processing conditions to produce waste materials with more predictable engineering behavior.

Advanced testing methods are another key trend. Researchers now combine conventional mechanical tests with microstructural, chemical, thermal, and environmental characterization. Techniques such as SEM, EDS, XRD, FTIR, TGA, BET surface area analysis, rheological testing, and durability testing are used to understand how waste materials interact with cement, bitumen, aggregates, and alkaline activators. In addition, leaching tests are becoming more important, especially for materials such as bauxite residue, mine tailings, waste rock, and acid mine drainage sludge, because these materials may contain alkaline components, sulfates, or trace metals. Recent evolutions in leaching assessment have highlighted the limitations of conventional batch testing methods, which often fail to replicate field conditions, including pH variation, compaction, and particle-size distribution. More representative approaches, including lysimeter testing, have been proposed to better simulate field conditions and provide more reliable data on contaminant release [216].

A further recent advance is the use of digital tools, modeling, and artificial intelligence. Machine learning is increasingly used to predict compressive strength, durability, rheological properties, optimum replacement levels, and long-term performance of waste-modified materials. These methods help reduce trial-and-error testing and support faster mix design. Software-based tools such as life-cycle assessment platforms, circularity assessment tools, and pavement performance models are also being used to compare environmental impact, carbon reduction, material utilization, and service-life performance. Recent studies show growing interest in combining machine learning with life cycle assessment to support more reliable sustainability decisions.

Overall, recent advancements demonstrate a clear shift from conventional waste utilization approaches toward more engineered, performance-driven, and sustainability-oriented systems. The integration of hybrid systems, advanced pre-processing techniques, optimized multi-component formulations, advanced characterization methods and testing, and latest data-driven design tools has significantly enhanced the feasibility of incorporating waste materials into civil engineering applications.

## 9. Conclusions and Future Perspectives

In conclusion, this review shows that the large amount of waste produced from industrial, mining, agricultural, and construction activities can play an important and practical role in many civil engineering applications, including concrete and cementitious systems, pavement engineering, soil stabilization, embankments, and base and sub-base infrastructure.

The review clearly demonstrates how selected waste materials are successfully used, taking into account each material’s physicochemical properties, mechanical behavior, and processing requirements. Industrial wastes such as fly ash and GGBFS are already widely used in cementitious applications, while recycled construction materials, RAP, waste plastics, biochar, agricultural ashes, and mining residues are also showing increasing potential in asphalt, geotechnical, and infrastructure works when properly treated and engineered.

One of the main findings is that waste utilization is not simply about replacing conventional materials, but about using each waste material where it performs best. Materials used as cement substitutes, fillers, aggregate replacements, stabilizers, or reinforcement agents behave differently depending on their reactivity, particle characteristics, durability, and environmental considerations. This means that choosing the right waste material for the right purpose is more important than focusing only on high replacement percentages.

Across the studies reviewed, properly processed waste materials have shown the ability to improve strength, durability, stiffness, resource efficiency, and sustainability. However, there are still limitations to consider, such as material variability, water demand, compatibility issues, environmental risks, and limited long-term validation, which still need careful attention.

Overall, the evidence suggests that waste materials are not just alternative materials for limited applications but valuable engineering resources with real potential for wider infrastructure use.

To further advance the utilization of waste materials in the civil engineering industry, the following research and development directions are recommended.

- Establish unified standard guidelines and specifications for materials such as mine tailings, bauxite residue, and recycled aggregates to support wider industry adoption. Address variability in waste materials through classification, processing, and quality assurance protocols.
- Conduct Large-scale field validation and long-term performance studies monitoring of durability, aging, and environmental performance of waste material utilized in construction applications. Advance leaching, toxicity, and long-term environmental impact evaluation, especially for mining-related wastes.
- It is recommended to further explore hybrid material systems using waste material in order to achieve balanced mechanical and durability properties.
- Further investigate more efficient and cost-effective pretreatment and activation techniques to improve reactivity and consistency of waste materials. The research areas can be expanded on converting waste into advanced materials (e.g., nano-silica from SCBA, engineered biochar, activated fillers) to improve performance in cementitious and asphalt systems.
- Incorporate life cycle assessment (LCA) and circular economy metrics into material selection and design processes to ensure environmental and economic sustainability.
- Upcoming research should emphasize the use of model-based models, machine learning, and optimization tools to forecast material performance and optimize mix designs, considering the variability of waste materials. In parallel, the application of digital tools such as simulation-based design, Building Information Modeling (BIM), and material databases can support efficient planning and decision-making.
- Strengthen collaboration between researchers, industry, and regulatory bodies to develop guidelines and policies that support the safe and efficient use of waste materials in the civil engineering industry.

In summary, the waste materials discussed in this paper demonstrate significant potential for sustainable utilization in civil engineering applications, offering both environmental and performance-related benefits when appropriately processed and designed. The future of sustainable construction lies in the effective transformation of waste into reliable, high-performance engineering materials, supported by advances in materials design, rigorous performance validation, and the development of standardized practices for large-scale implementation.

## Figures and Tables

**Table 1:** Characteristics of studies included in the review.

Waste Material Category	No. of Studies	Key Waste Materials	Engineering Applications	Representative References
Industrial waste materials	16	Fly ash	Supplementary cementitious material (SCM) in concrete. Primary precursor in alkali-activated & geopolymer binders. Mineral filler in asphalt mixtures. Soil stabilizer for road subgrade.	[6,13,14,15,16,17,18,19,20,21,22,23,24,25,26,27]
	17	Ground granulated blast furnace slag (GGBFS)	Latent hydraulic SCM in concrete and cementitious systems. Primary precursor in alkali-activated & geopolymer binders. Mineral filler in asphalt. Soil stabilizer for subgrade. Base and sub-base applications.	[10,26,27,28,29,30,31,32,33,34,35,36,37,38,39,40,41]
	18	Bauxite residue (red mud)	Secondary precursor in alkali-activated and geopolymer binder systems. Filler and mineral addition in concrete and asphalt. Binder modification in asphalt mixtures. Soil stabilizer for road subgrade.	[26,42,43,44,45,46,47,48,49,50,51,52,53,54,55,56,57]
Mining waste materials	17	Mine tailing	Filler and partial precursor in alkali-activated and geopolymer binder systems. Aggregate replacement in concrete and asphalt mixtures. Soil stabilizer for road subgrade. Embankment and fill applications. Base and sub-base construction	[18,58,59,60,61,62,63,64,65,66,67,68,69,70,71,72,73,74]
	13	Waste rock and overburden	Coarse aggregate replacement in concrete. Pavement geomaterial in road construction. Embankment and fill applications. Base and sub-base construction.	[75,76,77,78,79,80,81,82,83,84,85,86,87]
	5	Acid mine drainage sludge	Filler material in blocks and bricks. Soil stabilizer for road subgrade. Embankment and fill applications.	[88,89,90,91,92]
Plastic and polymer waste	15	Polyethylene (PE), Polypropylene (PP), Ethylene-vinyl acetate (EVA), Post-Consumer Vulcanized Rubber (PCVR)	Binder modification	[10,87,93,94,95,96,97,98,99,100,101,102,103,104,105]
Construction and demolition waste	10	Recycled concrete aggregate (RCA), demolition waste	Coarse/fine aggregate replacement in concrete. Aggregate in asphalt mixtures. Embankment and fill applications. Base and sub-base construction.	[106,107,108,109,110,111,112,113,114,115]
	12	Reclaimed asphalt pavement (RAP),	Recycled binder and aggregate in asphalt mixtures. Binder modifier in asphalt. Hybrid base course material. Base and sub-base construction	[106,108,110,111,112,115,116,117,118,119,120,121,122,123]
	7	Crushed masonry waste	Aggregate replacement in concrete and asphalt mixtures. Embankment and fill applications. Base and sub-base construction	[106,107,108,110,111,116,119]
Agricultural and biomass wastes	4	Biochar	Cement modifier and filler in concrete. Bitumen modifier and filler in asphalt mixtures.	[124,125,126,127]
	4	Rice husk ash (RHA)	Highly pozzolanic supplementary cementitious material (SCM) in concrete. Silica source in alkali-activated and geopolymer binder systems.	[19,56,128,129]
	3	Sugarcane bagasse ash (SCBA)	Supplementary cementitious material and partial cement replacement in concrete systems	[18,130,131]

**Table 2:** Oxide percentages of fly ash [134].

Oxide %	Class F	Class C
SiO_2_	53.5	40.2
Al_2_O_3_	27.3	17.5
Fe_2_O_3_	7.2	6.4
CaO	5.5	24.1
MgO	2.1	4.6
SO_3_	0.9	3.2
K_2_O	1.0	0.7
Na_2_O	0.4	0.6

**Table 3:** Chemical composition of GGBFS.

Reference	Chemical Composition %								
	SiO_2_	Al_2_O_3_	Fe_2_O_3_	CaO	MgO	SO_3_	Na_2_O	K_2_O	MnO
[33]	33.81	14.78	0.36	38.81	7.09	2.49	0.26	0.44	-
[31]	36.00	13.80	0.30	42.60	5.80	0.56	0.21	0.27	0.40
[32]	34.81	17.92	0.66	37.63	7.80	0.20	-	-	0.21
[34]	35.81	16.65	0.82	37.53	6.91	0.38	0.37	0.46	-
[35]	35.70	11.20	1.20	37.00	11.00	-	0.60	0.68	1.58
[36]	35.81	16.66	0.819	37.52	6.90	0.38	0.34	0.46	-

**Table 4:** Physical properties of bauxite residue [45,47].

Physical Properties	Value
Specific surface area (m^2^/g)	15–58
Particle size (mm)	0.075–0.005
Porosity ratio (%)	2.53–2.95
Specific gravity (kNm^−3^)	2.7–3.7
Moisture capacity (%)	80 (82.3–105.9)
Cation exchange capacity (mgg^−1^)	25–35 mg × 10^−2^ g
Density (kgm^−3^)	2700–2900

**Table 5:** Chemical Composition of Bauxite Residue.

Reference	Chemical Composition %					
	Fe_2_O_3_	Al_2_O_3_	TiO_2_	CaO	SiO_2_	Na_2_O
[48]	59.37	16.16	-	2.17	9.11	2.78
[49]	53.75	16.07	4.24	1.48	8.25	3.82
[50]	17.54	8.03	4.81	44.64	18.19	3.21
[51]	44.3	18.2	10.5	1.11	14.5	9.29
[52]	8.09	14.3	2.95	-	11.4	9.35
[53]	31.45	35.47	5.84	1.81	12.68	-
[54]	36.48	23.53	6.84	1.83	14.88	9.41
[55]	32.67	11.64	4.92	20.09	13.17	3.89

**Table 6:** Chemical composition of mine tailings.

Reference	Tailing Type	Chemical Composition %								
		CaO	SiO_2_	Al_2_O_3_	Fe_2_O_3_	SO_3_	P_2_O_5_	MgO	MnO	K_2_O
[68]	Iron	4.56	66.70	8.06	9.52	-	0.43	5.28	-	2.53
[69]	Gold	5.92	41.08	14.76	13.04	2.76	-	2.40	2.02	10.79
[70]	Copper	6.75	49.24	21.19	6.63	3.34	-	1.47	1.47	9.02
[71]	Molybdenum	3.36	71.84	11.47	1.85	-	-	-	0.05	7.32
[72]	Tungsten	-	44.83	18.39	11.85	10.94	-	-	-	3.62
[73]	Graphite	-	23.52	2.28	55.30	-	-	-	-	-
[74]	Coal gangue	0.29	40.42	46.11	0.56	0.01	0.51	0.10	-	0.23

**Table 7:** Chemical and physical properties of the waste rock/overburden.

Reference	Mining Type/Parent Rock	Physical Properties	Chemical/Mineralogical Composition
[80,81]	Coal Overburden	Highly heterogeneous, contains both fine and coarse particles	Dominated by SiO_2_ and Al_2_O_3_, Mineral phases with Quartz, kaolinite, and aluminosilicate minerals exhibit pozzolanic potential
[82]	Iron Overburden	Dense, hard, and angular	Fe_2_O_3_, SiO_2_, CaO, MgO, Al_2_O_3_
[83]	Copper Overburden	Moderate density and relatively low porosity, low moisture absorption	Al_2_O_3_, SO_3_, Sulfur, CaO, MgO, Na_2_O, K_2_O, P_2_O_5_, Cu, Au Chalcopyrite, Pyrite, Covelline, Chalcocite, Bornite, Cuprite, and Quartz, along with Kaolinite, sericite, etc.
[84]	Bauxite Overburden	Clay-rich, fine-grained overburden, variable composition with depth	Al_2_O_3_, SiO_2_, and Fe_2_O_3_. Dominated by kaolinite, gibbsite, goethite, minor hematite, anatase, quartz

**Table 8:** Physical properties of PE, PP, EVA, and PCVR.

References	Property	Polyethylene (PE)	Polypropylene (PP)	Ethylene-Vinyl Acetate (EVA)	Post-Consumer Vulcanized Rubber (PCVR)
[96,97]	Density (g/cm^3^)	0.91–0.96	0.90–0.92	0.93–0.95	1.10–1.25
[96,98]	Melting Point (°C)	110–130	160–170	75–100	Thermally degrades, does not melt due to cross-link structure
[98,99]	Flexibility	High	Moderate	Very High	High
[97]	Chemical resistance	High	High	Moderate	High
[99]	Water absorption	Very Low	Very Low	Low	Very Low

**Table 9:** Chemical characteristics of PE, PP, EVA, and PCVR.

Reference	Polymer	Main Elements	Structure Type	Key Features
[96]	Polyethylene (PE)	C, H	Linear chains	Flexible, Ductile
[97]	Polypropylene (PP)	C, H	Semi-crystalline (branched)	High strength, Fatigue resistance
[98]	Ethylene-Vinyl Acetate (EVA)	C, H, O	Copolymer (ethylene + Vinyl acetate)	Elastic, Adhesive
[103]	Post-Consumer Vulcanized rubber (PCVR)	C, H, S, O, Zn	Cross-Linked elastomeric network	Elastic, Durable, Abrasion-resistant

**Table 10:** Physical and chemical properties of RCA.

Reference	Property	Typical Range/Description	Key Influence
[110]	Density	2200–2600 kg/m^3^	Adhered mortar
[109,113]	Water absorption	4–10%	High porosity
[110]	Porosity	High	Microcracks, weaker interfacial transition zone (ITZ)
[108]	Shape & texture	Angular, rough	Crushing process
[106]	Strength	Moderate–low	Weak mortar layer
[111]	Chemical composition	CaO, SiO_2_, Al_2_O_3_	Cement paste
[107]	pH	>10	Alkaline hydration products

**Table 11:** Physical and chemical properties of RAP.

Reference	Property	Typical Range/Description	Key Influence
[108]	Density	2300–2500 kg/m^3^	Aggregate composition
[112]	Binder content	3–7%	Residual bitumen
[110]	Water absorption	Low–moderate	Bitumen coating
[108]	Texture	Smooth, coated	Asphalt film
[111]	Stiffness	High	Binder aging
[112]	Chemical composition	Hydrocarbons & minerals	Bitumen & aggregates

**Table 12:** Physical and chemical properties of crushed masonry waste.

Reference	Property	Typical Range/Description	Key Influence
[110]	Density	1800–2400 kg/m^3^	Ceramic structure
[107]	Water absorption	8–20%	High porosity
[108]	Porosity	Very high	Fired clay
[110]	Strength	Low	Brittle material
[111]	Chemical composition	SiO_2_, Al_2_O_3_	Clay minerals
[110]	Sulfate content	Variable	Gypsum contamination

**Table 13:** Physical and chemical characteristics of biochar.

Reference	Property	Typical Range
[124]	Surface area (m^2^/g)	50–500
[125]	Density (g/cm^3^)	0.3–0.6
[125]	Carbon content %	50–90
[124]	Structure	Highly porous
[125]	PH	7–10

**Table 14:** Physical and chemical characteristics of Rice Husk Ash.

Reference	Property	Typical Range
[128]	Surface area (m^2^/g)	50–500
[128]	Density (kg/m^3^)	500–700
[129]	Silica content (%)	85–95
[128]	Carbon content (%)	1–5
[129]	Structure	Amorphous silica

**Table 15:** Physical and chemical properties of SCBA.

Reference	Property	Typical Range
[131]	Density (kg/m^3^)	600–900
[130,131]	Silica (SiO_2_) content %	60–80
[131]	Alumina (Al_2_O_3_) content %	5–15
[130]	Iron Oxide (Fe_2_O_3_) content%	1–5
[131]	Structure	Porous, irregular

**Table 16:** Summary of global annual availability, key material features, and primary environmental concerns of major waste materials used in civil engineering.

Waste Material	Global Availability/Annually	Key Material Features	Main Concern	Reference
Fly ash	500 million tonnes	Fine, spherical particles (10–50 µm) with high specific surface area. Rich in amorphous aluminosilicate phases (SiO_2_, Al_2_O_3_, Fe_2_O_3_) with variable CaO content (Class F and Class C). Exhibits pozzolanic or cementitious behavior.	Variability in composition and quality; potential leaching of heavy metals (Pb, Ni, Zn). Environmental and health risks are associated with improper disposal and airborne dispersion.	[6,13,15,16]
Ground granulated blast furnace slag	530 million tonnes	Fine powder with predominantly amorphous (glassy) structure. Rich in CaO–SiO_2_–Al_2_O_3_; exhibits latent hydraulic reactivity. Moderate to high surface area.	Variability in chemical composition depends on raw materials and production process. Requires proper activation (e.g., alkali or cement) for reactivity.	[29]
Bauxite residue	150 million tonnes	Fine-grained, highly alkaline residue (pH = 10–12.5) with high specific surface area. Rich in Fe_2_O_3_, Al_2_O_3_, and other metal oxides. Contains reactive mineral phases with potential for reuse in cementitious, geopolymer, and filler applications.	High alkalinity, heavy metal, and potential radioactive content. Environmental risks from leaching and dust dispersion, and the need for pre-treatment or stabilization before safe utilization.	[45,46,47]
Mine tailing	7–14 billion tonnes	Fine-grained, angular, and mineralogically diverse waste with variable silica. Alumina, iron, and calcium content depend on the ore source. Moderate bulk density with potential use as filler, aggregate, or geopolymer precursor when properly processed.	Potential acid mine drainage, sulfide oxidation, heavy metal leaching. Chemical contamination and highly variable composition require careful environmental assessment and stabilization.	[66,67]
Waste rock and overburden	50 billion tonnes	Highly variable coarse-to-fine materials depending on parent geology. May include aluminosilicates, iron oxides, sulfides, or clay-rich phases. Often mechanically stable with potential use in embankments, aggregates, backfill, or sub-base applications.	Large-volume land disturbance, slope instability. Acid generation has potential in sulfide-bearing materials. Site-specific heterogeneity, and environmental risks including land degradation and water contamination.	[75,76]
Acid Mine Drainage Slag	averages of 9500 dry tonnes/site/year (Approx)	Fine-grained metal-rich sludge containing Fe, Al, Mn oxides/hydroxides, and sulfate-bearing phases. Potential for stabilization or secondary reuse after treatment.	Hazardous metal content. Contaminant leaching risk, disposal burden, and variability depending on AMD chemistry and treatment process.	[88,89]
Polyethylene	100–120 million tonnes	Thermoplastic polymer with low density and semi-crystalline structure. Hydrophobic and high moisture resistance. Low melting point (~110–130 °C), more suitable for asphalt reform. Improves flexibility and ductility in composites.	Non-biodegradable. Weak bonding with cement. Microplastic pollution risk. Need structured processing for uniform distribution.	[137]
Polypropylene	80–90 million tonnes	Semi-crystalline thermoplastic (higher stiffness than PE). High tensile strength and fatigue resistance. Generally used for fiber reinforcement. Improves crack resistance and toughness.	Poor interfacial bonding with the cement. UV degradation over time. Non-biodegradable Recycling challenges.	[137]
Ethylene Vinyl Acetate (EVA)	5–7 million tonnes	Flexible copolymer with rubber-like behavior. High elasticity and impact resistance. Good adhesion, mainly in bitumen modification. Improves the flexibility and durability of asphalt.	Thermal instability (high temperatures). Limited recycling methods. Performance varies with the ratio. Compatibility issues in mixtures.	[137]
Post-Consumer Vulcanized Rubber (PCVR)	1–1.5 billion tonnes	High elasticity, abrasion resistance, and durability Highly cross-linked elastomeric structure Contains NR, SBR, BR, carbon black, sulfur, and additives Processed into crumb rubber or ground tire rubber (GTR) powder.	Non-biodegradable High resistance to degradation End up in landfills and stockpiles, resulting in a fire hazard Microplastic formation and ground and water contamination	[102,105]
Recycled Concrete aggregate	>3 billion tonnes	Crushed concrete with mixed mortar. Higher porosity and water absorption (than natural aggregates). Includes residual cementitious parts. Can contribute to partial self-cementing.	Reduced strength due to a weak interfacial transition zone (ITZ). High variability depending on source. Requires processing (washing/pre-treatment) Durability concerns.	[107,109,138,139]
Reclaimed asphalt pavement	100 million tonnes	Contains aged bitumen and aggregates. High stiffness due to binder oxidation. Reusable in asphalt mixtures. Reduces demand for virgin materials.	Reduced flexibility due to aged binder. Requires rejuvenators for performance. Variability in binder content Mixing and compatibility issues.	[138]
Crushed masonry waste	~3 billion tonnes (C&D waste)	Composed of bricks, tiles, and ceramics. Lower density and higher porosity. Good thermal insulation properties. Suitable for non-structural applications.	Lower compressive strength, high water absorption. Variability depends on the source. Limited structural use.	[106]
Biochar	0.5–1 million tonnes (increasing)	Carbon-rich porous material from pyrolysis. High surface area and water retention. Lightweight and improves soil properties. Improves microstructure in some composites.	Highly variable depending on feedstock. Limited long-term performance data. Potential contamination. Requires controlled production.	[125]
Rice Husk Ash	20 million tonnes	High amorphous silica (~85–95%). Fine particles with high pozzolanic activity. Improve durability and reduce permeability. Enhance long-term strength.	Quality depends on burning conditions. Unburnt carbon reduces. Handling difficulties due to fineness. Requires processing.	[128,129]
Sugarcane Bagasse Ash	10–15 million tonnes	Silica-rich pozzolanic material. Improves workability and durability. Enhances long-term strength. Suitable for cement and soil stabilization.	High variability due to combustion conditions. Presence of unburnt carbon. Requires grinding and sieving. Limited standardization.	[130,131]

**Table 17:** SCM behavior and performance in concrete.

Material	Behavior Type	Optimum Replacement (%)	Key Quantitative Finding	Durability (Permeability/Chloride)	Key Limitation	Reference
Fly ash	Pozzolanic	15–35	SAI increased from 7.4% to 12.5% (3 d) to 92.13% (28 d)—indicates strong long-term pozzolanic activity	Reduced permeability and durability	Slow early age reaction	[22]
GGBFS	Latent hydraulic	20–40	Compressive strength increased from 130 to 140 MPa at 90 days (8% increment)	Excellent chloride and sulfate resistance	Excessive replacement leads to reduced strength	[29,37]
Rice husk ash	Highly pozzolanic	30	UHPC compressive strength reached 182 Moa at 28 days (51% increment)	Strong pore refinement and reduced permeability	Sensitive to processing conditions and burning	[56]
SCBA	Pozzolanic	5	5 wt.% SCBA improved mortar strength, Si NP further improved strength by improving hydration	Improves durability and microstructure	High variability in ash quality and composition	[140]

**Table 18:** Materials in alkali-activated and geopolymer systems.

Material	Role in System	Strength Development	Key Quantitative Findings	Key Advantage	Reference
Fly ash	Primary precursor	Moderate early strength, high later age strength	5% addition of slag increased 28-day compressive strength by 16% while reducing flowability 8.5%	Widely available and suitable for geopolymer production	[23]
GGBFS	Primary precursor/Activator enhancer	High early strength	Fly ash, GGBFS, and metakaolin systems achieved 62 MPa compressive strength in 28 days	Accelerates geopolymerization and matrix densification	[39]
Bauxite residue	Secondary precursor/Soil Precursor	Moderate strength gain when activated	content. NaOH-activated BR soil mixtures achieved UCS of 2.23–3.05 MPa after 7 days	Enhances soil stabilization and pavement performance	[141]
Mine tailings	Precursor in geopolymer systems	Moderate to high when activated	Mixtures containing 70% water tailings achieved 31 MPa at 28 days, and geopolymer bricks reached 49 MPa after 90 days	Enables waste immobilization	[18,58]
Rice husk ash	Silica-rich precursor	Moderate to high strength development	Achieved compressive strengths of 34 MPa	High reactive silica and gel formation	[142]

**Table 19:** Filler and aggregate replacement materials in concrete.

Material	Primary Role	Optimum Replacement	Porosity Impact	Key Quantitative Finding	Key Benefit	Reference
Bauxite residue	Cement/fine aggregate replacement	10	May increase if not optimized	Compressive strength comparable to or slightly higher than conventional concrete; strength decreases beyond 10–15% replacement	Waste reuse reduces cement demand	[143]
Mine tailings	Fine aggregate replacement	10–35	Variable	Replacement up to 60% achieved strength compared to control concrete	Abundant, low-cost material	[59,60]
AMD sludge	Filler (blocks/bricks)	N/A	Variable	Surface area—31.5 m^2^/g and volume 0.41 cm^3^/g—Suitable for value added products	Waste stabilization	[91]
RCA	Coarse aggregate replacement	15–30	Higher porosity	15–60% RCA reduced compressive strength by 12–46% and flexural strength by 20%	Reduces natural aggregate use	[144]
Crushed masonry	Fine aggregate replacement	15–20	Increased porosity	10–20% replacement achieved 27–29 MPa compressive strength, 50–75% reduced strength by 50%	Circular economy benefits	[118]

**Table 20:** Binder modification using waste materials.

Material	Incorporation Method	Main Benefit	Key Quantitative Finding	Low-Temp Performance	Key Limitation	Reference
Polyethylene (PE)	Wet/Dry	Increased stiffness	Softening point increased by 19%	Reduced flexibility	Poor compatibility	[10,146]
Polypropylene (PP)	Wet/Dry	High strength	Optimum dosage 0.6%	Brittle behavior	Phase separation	[101,147]
EVA	Wet	Good compatibility	Softening point increased 25%	Moderate	Higher cost	[101,146]
Post-Consumer Vulcanized Rubber (PCVR)	Wet/Dry	Improved elasticity and fatigue resistance	Rutting resistance increased 7 times; fatigue life increased 12.6 times	High	Increased viscosity and storage stability issues	[102]
Biochar	Dry	Aging resistance	Critical rutting temperature 65.2 to 70.1 °C	Slight reduction	Limited standardization	[126]
Bauxite residue	Dry	Stiffness improvement	3–15% filler replacement improved softening point, viscosity, and rutting resistance	Limited data	Reduced ductility	[26]

**Table 21:** Mineral fillers and aggregate replacement in asphalt.

Material	Role	Optimum Content %	Key Quantitative Finding	Main Benefit	Key Limitation	Reference
Fly ash	Filler	4–6	Marshall stability increased from 13.56 to 19.48 kN; OBC reduced from 5.21% to 4.8–4.9%	Improved strength, Reduced bitumen demand	Excessive usage affects workability	[20,24].
GGBFS	Filler	3	Marshall stability increased from 16.16 to 19.16 KN	Rutting resistance and stability improved	Performance decreased after the optimum content	[10,40]
Bauxite residue	Filler	3–7	Permanent deformation reduced from 6.1% to 3.5–5.31%	Rutting resistance improved	Moisture resistance decreased (88.57–82.28 RMS)	[26,149].
RCA	Aggregate	<30	Increased Marshall stability upto 45%	Conserve natural aggregates	Higher air voids and moisture susceptibility at high contents	[110,150]
Crushed masonry	Aggregate	10–30	Marshall properties are acceptable <30% replacement	Improved aggregate interlock, sustainable	Higher binder demand and moisture susceptibility	[119]
Mine tailings	Filler	3–7	Softening point increased by 14.6%,	Stiffness and rutting resistance improved	Ductility and moisture resistance were reduced	[61,62].

**Table 22:** Recycled asphalt and hybrid systems.

Material/System	Role	Key Performance	Main Limitation	Reference
RAP	Binder & aggregate	45–60% binder blending efficiency, improves rutting resistance and stiffness	Increased brittleness and cracking	[117,120]
RAP + PE/PP/EVA	Polymer-modified binder	Optimum 3–8 wt.%, improved rutting resistance and durability	Reduced low-temperature flexibility	[121]
RAP + Biochar	Binder modifier	Increase aging resistance and durability	Limited long-term field validation	[118]
RAP + GTR (PCVBR)	Layered system	Optimum 10–20%, increased softening point 18.7%, and increased shear strength 45.9%	Reduced ductility (30.9%), high mixing and compaction temperatures.	[110,122]
RAP + RCA/Crushed masonry	Hybrid base	Improves resource efficiency	Variability in mechanical performance	[119]

**Table 23:** Soil stabilization using waste materials.

Material	Optimum Content	Key Performance	Main Limitation	Reference
Fly ash	Up to 40%	UCS increased 40–48%, CBR increased 52–55%, swelling reduced 42–48%, and reduced compressibility 36–40%	Fly ash class decides performance, often mixed with lime/cement	[21,25]
GGBFS + PG	2.5% PG	UCS increased 40%, Cost reduced 43%, and CO_2_ emissions reduced 37%	Alkali activation required	[38,41]
Bauxite residue	2%	UCS increased 426%, BTS increased 167%	High Alkalinity, optimization required	[151]
AMD sludge	>3%	As stabilization > 95%, Pb > 90%, Zn > 70%	Long-term field validation is required	[92]
Mine tailings	20% MT + 4% Lime	Increased UCS from -40.1 to 260.5 kN/m^2^	Lime activation and curing are required	[63]

**Table 24:** Waste materials in embankment and fill applications.

Material	Application	Key Performance	Main Limitation	Reference
Waste rock	Structural fill	High strength and good stability	Acid generation potential	[85]
Magnesite Mine tailings	Structural fill	UBC 150.5–762.6 kPa; modulus of subgrade reaction 26,140–90,960 kN/m^3^	Leaching control required	[61,64]
RCA	Subgrade stabilization	20% RCA: MDD increased by 6.35%, UCS increased 175.7%, and shear strength increased 175.5%,	Slight CBR reduction −4.1%	[110,152].
Crushed masonry	Fill/Subbase	Suitable for engineered filling and subbase	Variable water absorption and strength	[119]
AMD sludge	Structural fill/Soil amendment	Potential for reuse after treatment	Leaching control required	[92]

**Table 25:** Waste material in base and sub-base applications.

Material	Optimum Content	Key Performance	Main Limitations	Reference
Waste rock + Crumb Rubber	0.5–2% Rubber	Cohesion 25.3–41.6 kPa; improved friction angle and shear stiffness	High rubber content reduces cohesion	[87]
RCA	>3% Cement	7-day UCS ≥ 2 MPa; 5–7% cement achieved 3–4.5 MPa; <14% weight loss after 12 wet–dry cycles	Cement treatment required	[153]
RAP	50–55%	Soaked CBR increased from 32% to >100%	Higher RAP content may reduce durability	[123]
Fly ash + Lime + GGBFS	88% FA + 3% Lime + 9% GGBFS	Met pavement base strength requirement through cementitious gel formation	Activators required	[26]
Zinc Mine tailings	Cement stabilized	Dry density 18.62 kN/m^3^; CBR 11%; friction angle 34°; modulus 18.17 MPa; reduced aggregate thickness by 170 mm	Cement stabilization required	[65]

**Table 26:** Summary of GWP reduction and cost benefits of waste materials in construction applications.

Waste Material	Application	GWP Reduction (%)	Cost Reduction	Ref
Fly ash (class F)	50% Cement replacement in concrete	54%	-	[157]
GGBFS	65% Cement replacement in concrete	61%	Binder costs lower than cement-only systems	[157]
RHA	10–35% cement replacement in concrete	25% CO_2_ emission reduction	65% rise in the cost efficiency, unit cost 43–51% lower than cement	[158]
SCBA	10–30% cement replacement in mortar	16% GWP reduction at 20% replacement (21 °C curing); 32% at 45 °C curing	18.5% cost reduction at 30% replacement	[159,160]
Biochar	3–5% cement replacement in concrete	12–20%	The cost of concrete can be reduced by up to 0.66%	[161,162]
RCA	100% replacement of natural fine aggregate	64% reduction	30% lower production cost	[163,164]
Crumb rubber	5–20% bitumen modifier in asphalt (wet process)	71.9% CO_2_ reduction (recycling vs. landfill/incineration), 2.23% reduction in asphalt mix	16.1 million USD/yr resource savings (Australia)	[166]
RAP	10–30% total aggregate mass in HMA	6.8% reduction	Up to 14.1% production cost reduction	[167]
polyethylene (LDPE and HDPE)	2–8% by weight of bitumen (wet process)	8.6–15.6% reduction compared to virgin polymers (SBS, LDPE, HDPE)	Cost-effective and cheaper than virgin polymers	[168]
Bauxite residue	Up to 25% cement replacement	26%	-	[165]

**Table 27:** Replacement of natural materials using waste materials in construction.

Waste Material		Natural Material Replaced	Typical Replacement Level	Resource Category Conserved							Ref
				Limestone	Clay	Energy Resources	Mineral Fillers	Natural Aggregates	Natural Sand	Petroleum Resources (Crude Oil)	
Fly ash		Portland cement	15–30%	√	√	√					[170]
GGBFS		Portland cement	30–50% (up to 70%)	√	√	√					[29]
Bauxite residue		Portland cement/mineral filler	5–40%, up to 50% filler substitution, 10–15% (optimal SCM), up to 30% (blended cement), 12–25% (concrete), up to 50% (bricks/ceramics)	√	√	√	√			√	[5]
Mine tailings	Iron	Road base/coarse & fine aggregates/sand, and Portland cement in concrete	10–20% (road base), 74% coarse aggregate, 12% fine aggregate, 40% as sand, and 5% as cement in concrete	√	√	√		√	√		[172,173,174,175]
	Gold	Sand/Portland cement	Up to 25–50% (sand), 20% (cement)	√	√	√			√		[178,179]
	Copper	Sand/filler/Portland cement	35% (sand), 5–10% (cement), F/A ratio 0.3–1.2 (filler)	√	√	√	√		√		[180,181]
	Molybdenum	Road base/sand Portland cement	15% (road base), up to 50% (sand replacement), 10% cement					√	√		[182,183,184]
	Tungsten	Cement/cement filler	20–30% (cement replacement), 50–75% (cement filler substitution)	√	√	√	√				[185]
	Graphite	Fine aggregates	50%						√		[186]
	Coal gangue	Portland cement/cement filler	20–30% (cement replacement), 50–75% (cement filler substitution)	√	√	√	√				[187]
Waste rock & overburden		Natural aggregates (coarse & fine)/pavement base materials/cement (tiles)s	Up to 40% (concrete), up to 100% (base/subbase applications), partial fine aggregate replacement (0–5 mm fraction)					√	√		[5]
Acid mine drainage sludge		Clay (brick manufacturing)/adsorbent materials (for wastewater treatment),	20% (brick production), full utilization in treatment systems (pellets)		√						[90]
Plastic wastes (polyethylene, polypropylene)		Bitumen modifier/aggregates	6–10% as bitumen modifier, 50–75% as aggregate in lightweight concrete					√		√	[9,10]
EVA		Bitumen modifier	3–7%							√	[11]
Recycled concrete aggregate (RCA)		Natural aggregates	Fully or partially replaces natural aggregate					√			[177]
Reclaimed asphalt pavement (RAP)		Aggregates/bitumen	15–30%					√		√	[176]
Biochar		Cement/bitumen modifier/filler	1–3% (optimum), up to 5% in concrete; 5–10% in bitumen	√	√	√				√	[127]
Rice husk ash (RHA)		Portland cement	5–20%	√	√	√					[7]
Sugarcane bagasse ash (SCBA)		Portland cement	10–20%	√	√	√					[8]

## Data Availability

No new data were created or analyzed in this study. Data sharing is not applicable to this article.

## Supplementary Materials

The following supporting information can be downloaded at: https://www.mdpi.com/article/10.3390/ma19143154/s1. PRISMA-ScR checklist [217].
